# Logic-gated T cell engager with mutant IL-2 potentiates antitumor responses without cytotoxicity in solid tumor models

**DOI:** 10.1172/JCI204657

**Published:** 2026-11-16

**Authors:** Shijie Li, Nan Li, Xiangming Liu, Yueqi Cai, Mukai Wang, Huimin Wang, Yang-Xin Fu, Zaopeng Yang

**Affiliations:** 1 Changping Laboratory, Beijing, China.; 2 Center for Cancer Biology, School of Basic Medical Sciences, and; 3 State Key Laboratory of Molecular Oncology, Tsinghua University, Beijing, China.; 4 Institute for Molecular and Cellular Therapeutics, Chinese Institutes for Medical Research, Beijing, China.; 5 School of Basic Medical Sciences, Capital Medical University, Beijing, China.

**Keywords:** Immunology, Oncology, Cytokines, Drug therapy, T cells

## Abstract

T cell engagers (TCEs) have achieved transformative success in B cell malignancies but show limited efficacy in solid tumors with severe toxicity. We propose that TCR signaling without IL-2 might limit the function of tumor-infiltrating lymphocytes (TILs). Here, we showed that insufficient IL-2 signaling is a key bottleneck for sustaining TCE-induced T cell function. Although exogenous IL-2 enhances TCE efficacy, its systemic administration causes more severe toxicity. We engineered a protease-activatable dual-variable domain (DVD) as a pro-antibody to reduce αCD3 off-tumor toxicity. Furthermore, we designed 3 additional mutations for IL-2 (3E) with markedly reduced receptor affinity, which remained inactive in the periphery. By coengineering the DVD and 3E into a single pro-drug molecule (DVD-3E), we achieved conditional IL-2 and TCR cosignaling strictly dependent on *cis* CD3 engagement within the tumor microenvironment (TME) upon protease cleavage. This logic-gated design restricted both TCE and cytokine activity to the TME, enabling potent antitumor responses without systemic toxicity. Mechanistically, DVD-3E enhanced not only the persistence and effector function of preexisting intratumoral T cells but also expanded TCF1^+^ TILs, mitigating exhaustion and enabling durable systemic immunity. These findings define a new design principle for logic-gated TCEs that achieve a favorable balance between efficacy and safety, offering a strategy to overcome current therapeutic resistance.

## Abstract 2

A new T cell engager uses tumor-controlled IL-2 signaling to enhance cancer treatment while reducing toxicity.

## Graphical abstract

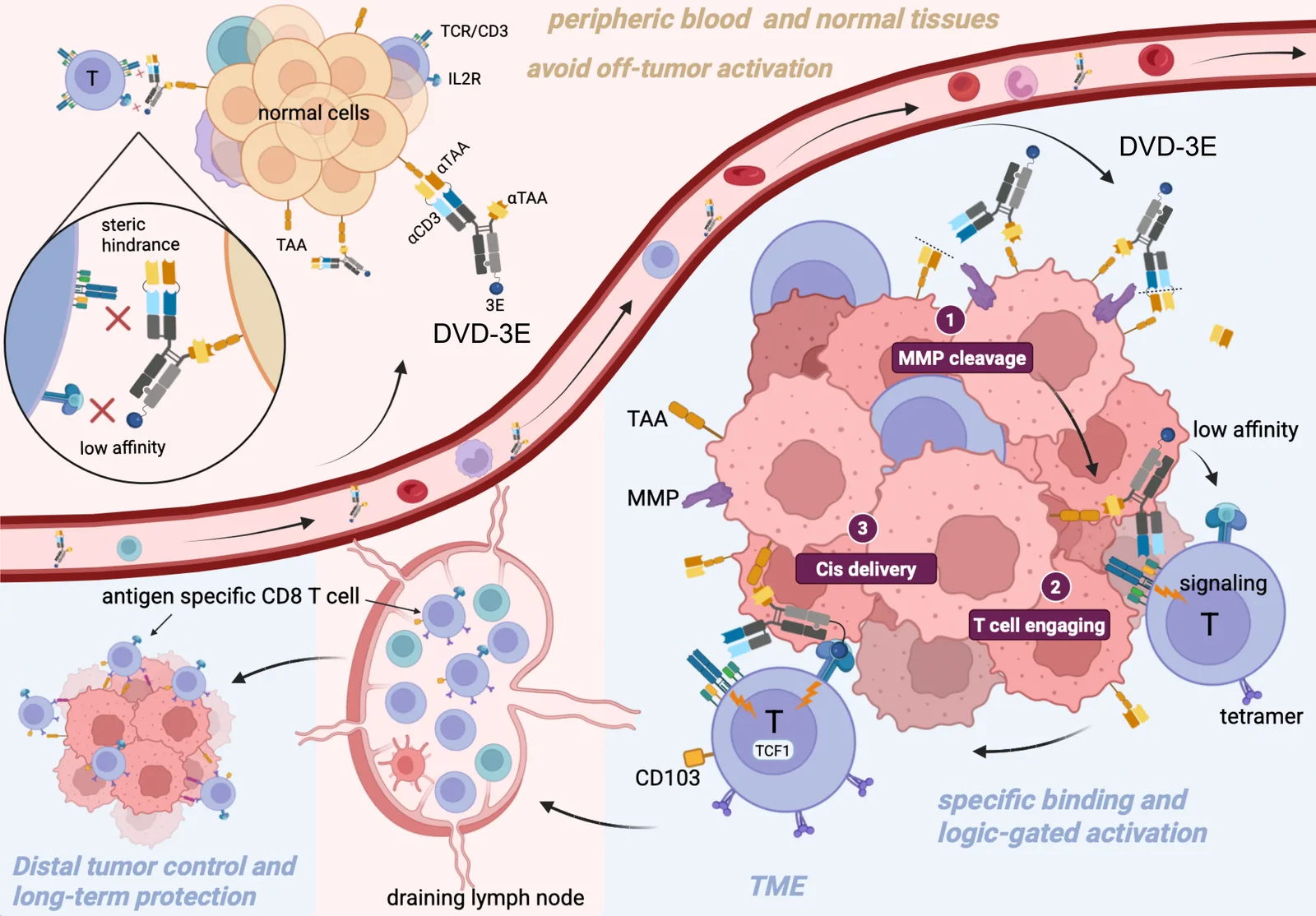

## Introduction

Immunotherapy has reshaped cancer treatment by harnessing the immune system to eradicate malignant cells (1). Among emerging modalities, bispecific T cell engagers (TCEs) represent a powerful engineering approach that redirects tumor-infiltrating lymphocytes (TILs) to recognize and eliminate tumor cells in an MHC-independent manner (2–4). Through simultaneous binding to CD3 on T cells and a tumor-associated antigen (TAA), TCEs can bypass antigen presentation defects and rapidly initiate target cell lysis (2). Several TCE agents, including blinatumomab (5), mosunetuzumab (6), and teclistamab (7), have achieved remarkable clinical success in B cell malignancies, with multiple FDA approvals since 2014 (3). However, their efficacy in solid tumors remains limited with severe toxicity (8, 9).

One key barrier lies in the dysfunctional state of TILs (10). Chronic antigen-only exposure drives rapid T cell exhaustion (11, 12), marked by diminished proliferative capacity, impaired cytotoxicity, and sustained expression of inhibitory receptors such as PD-1 and TIM-3 (13). In addition, conventional TCEs deliver only the primary activation signal (CD3 engagement) without sufficient costimulation, leading to transient activation followed by functional collapse (14, 15). Inspired by CAR engineering (16), several groups have attempted to incorporate costimulatory domains or accessory modules into TCE scaffolds to improve persistence (17); however, these modifications have not translated into consistent clinical benefit. We hypothesize that cytokine support (signal 3), particularly IL-2, is essential for sustaining T cell expansion and survival and for rejuvenating dysfunctional TILs. However, following the first signal, TILs have access to only limited amounts of endogenous IL-2, which is rapidly consumed. While *cis* delivery of exogenous IL-2 can synergize with TCE activity, such fusion proteins might exhibit even more toxicities (18, 19).

To achieve a balance between efficacy and safety, we designed a modular fusion protein that integrates tumor-restricted activation with localized cytokine signaling through *cis* delivery. The construct comprises 2 synergistic components: (a) a protease-activatable dual-variable domain (DVD) to shield CD3 (20), named Pro-TCE, that remains inert in circulation and becomes active only after tumor-localized proteolytic cleavage and (b) a mutant IL-2 (3E) variant with markedly reduced receptor affinity to both α and β, whose signaling is contingent on colocalization with the cleaved DVD-3E complex. This design ensures a logic-gated response, confining T cell engagement and cytokine activation to the tumor microenvironment (TME), thereby enhancing safety and efficacy.

In preclinical models, this DVD-3E fusion elicited potent antitumor activity, mitigated T cell exhaustion, and minimized systemic cytokine release, thereby identifying a therapeutic sweet spot between efficacy and safety. Here, we introduce a design principle for safer and more effective TCEs in solid tumors, with broad translational potential and synergy with existing immuno-oncology modalities.

## Results

### Introduction of IL-2 sustains T cell activity and boosts TCE-mediated tumor regression.

Although TCEs have demonstrated partial antitumor efficacy (21), their ability to sustain tumor control in solid tumors remains limited (Figure 1A), even under intensified dosing regimens, and treatment is frequently accompanied by body weight loss (Supplemental Figure 1A; supplemental material available online with this article; https://doi.org/10.1172/JCI204657DS1). In coculture assays of PBMCs and tumor cells with TCE, repeated tumor rechallenge rapidly rendered PBMCs incapable of mediating effective tumor cell killing (Figure 1B), indicating progressive loss of effector function. To evaluate the effects of TCE on T cell responses within the tumor immune context, exhausted T cells (Tex) were generated by repeated in vitro stimulation with anti-CD3 and anti-CD28 Dynabeads, mimicking the dysfunctional state of T cells in the TME. Bulk RNA-seq revealed marked downregulation of *IL2* and *IFNG* expression in Tex cells (Figure 1C) and cytokine-related pathways (Figure 1D). These Tex cells exhibited elevated expression of exhaustion markers and reduced IL-2 (Figure 1E), IFN-γ, and TNF-α production (Supplemental Figure 1B), suggesting that Tex cells lost the ability to secrete cytokine upon repeated stimulation.

We propose that lack of sufficient IL-2 might limit CD3-driven T cell function. Thus, we tested if exogenous IL-2 could have better cell expansion on T cells (Supplemental Figure 1C). Indeed, IL-2 allowed better T cell survival and expansion. We further tested if Tex cells lack sufficient IL-2 to sustain their cytotoxicity. In an in vitro killing assay, the addition of IL-2 substantially enhanced TCE-mediated tumor cell lysis by T cells compared with treatment with TCE or IL-2 alone (Figure 1F). Greater differentiation was observed in IFN-γ secretion (Figure 1G), further supporting that additional IL-2 sustains the cytotoxicity of T cells. Next, we engineered a trispecific fusion protein by incorporating IL-2 into the TCE framework, termed TCE-IL2. TCE-IL2 demonstrated substantially enhanced tumor-killing activity compared with TCE alone (Figure 1H). To further validate its efficacy in vivo, we established an MC38-CLDN tumor model in hCD3EDG-knockin (KI) mice and treated the animals with TCE and TCE-IL2 following tumor inoculation. TCE-IL2 revealed a more powerful tumor control ability than TCE (Figure 1I). However, TCE-IL2 induced substantial peripheral toxicity, as evidenced by substantial body weight change (Figure 1J) and cytokine release, especially IFN-γ (Figure 1K) and TNF-α (Supplemental Figure 1D).

### The pro-body DVD format enables tumor-restricted TCE activation and reduces systemic toxicity.

To reduce the nonspecific peripheral activation observed with conventional TCE, we designed a Pro-TCE in a modified DVD format (20), which represents an alternative 2+1 design (22). This protease-activated TCE shields the inner CD3-binding domain with an outer tumor-targeting anti TAA domain, connected via short MMP cleavable linkers (Figure 2A). Surface plasmon resonance (SPR) analysis revealed marked differences in CD3 binding affinity between DVD and conventional TCE formats (Figure 2B). Consistently, in vitro studies demonstrated that using cleavable linkers effectively reduced CD3 binding to T cells while enabling affinity restoration following MMP cleavage (Figure 2C and Supplemental Figure 2A). Moreover, incorporation of the outer TAA-binding domain further enhanced tumor antigen recognition of the DVD molecule (Supplemental Figure 2B). In vitro coculture experiments demonstrated that the DVD provides superior masking of CD3 activity and can regain its T cell activation (Figure 2D) and killing function (Figure 2E) upon MMP cleavage. In vivo studies further confirmed that treatment with an equimolar dose of DVD and conventional TCE achieved comparable antitumor efficacy (Figure 2F), while DVD treatment markedly reduced systemic toxicity (Figure 2, G and H). To further establish the requirement of protease-mediated activation for DVD function, we evaluated its antitumor activity in the presence of the broad-spectrum MMP inhibitor GM6001. Cotreatment with GM6001 substantially attenuated the therapeutic efficacy of DVD in MC38-CLDN tumor-bearing mice (Figure 2I), supporting the critical role of MMP-dependent proteolytic activation in enabling DVD activity. Moreover, replacement of the MMP cleavage site with a noncleavable G4S linker markedly reduced antitumor activity (Supplemental Figure 2C). Together, these findings demonstrate that protease-dependent activation is essential for the therapeutic activity of the DVD platform and enables tumor-restricted TCE activation with improved safety profiles.

### 3E synergizes with CD3 engagement to enable logic-gated T cell activation.

We next evaluated the impact of fusion site on protein behavior by attaching WT IL-2 to 3 distinct C-terminal positions within the DVD framework. Among these constructs, only the format in which IL-2 was fused to the anti-CLDN chain exhibited acceptable protein quality (Supplemental Figure 3, A–C), although all formats retained antigen-binding capacity (Supplemental Figure 3D) and residual IL-2 activity (Supplemental Figure 3E). Based on these results, we selected the anti-CLDN–linked IL-2 construct (hereafter referred to as DVD-IL2) and found that it substantially enhanced antitumor efficacy (Supplemental Figure 3F) but also reintroduced marked systemic toxicity (Supplemental Figure 3, G and H). To overcome IL-2–associated systemic toxicity while preserving its therapeutic potential, we sought to engineer an IL-2 variant whose activity would remain latent and only be functional upon CD3 engagement. Among the 3 IL-2 receptor subunits (α, β, and γ), IL-2Rα exhibits the highest binding affinity for IL-2 (23). Thus, we prioritized reducing IL-2’s affinity for IL-2Rα as a key strategy. Specifically, we introduced glutamic acid (E) substitutions at residues K35, R38, and K43, 3 key IL-2 residues that mediate contact with IL-2Rα, generating the IL-2 variant termed 3E. Structural modeling suggested that these substitutions disrupt the electrostatic interactions required for IL-2 engagement with the IL-2Rα interface (Figure 3A). To assess the functional impact of these mutations, human PBMCs were stimulated with either WT IL-2 or the 3E variant. Compared with WT IL-2, 3E induced an approximately 1,000-fold reduction in STAT5 phosphorylation (pSTAT5), confirming that 3E effectively reduces IL-2 signaling in T cells (Figure 3B). We subsequently incorporated the 3E variant into the DVD format to generate DVD-3E (Figure 3C). Both in vitro binding assays and AI-based structural modeling indicated that the 3E mutations not only abolished IL-2 binding to IL-2Rα (Figure 3D), but also unexpectedly impaired IL-2Rβ binding (Figure 3E and Supplemental Figure 3I). Reduced receptor activation by DVD-3E was further confirmed using an IL-2–responsive HEK reporter system (Figure 3F). Since pSTAT5 is a central signaling event downstream of IL-2 receptor engagement, and prior studies suggest *cis*-binding can partially rescue IL-2 signaling for low-affinity variants, we investigated whether CD3 engagement could restore 3E activity. We generated matched DVD, DVD-IL2, and DVD-3E constructs before and after MMP-mediated activation (Figure 3G). Our results showed that 3E-mediated pSTAT5 signaling was largely dependent on CD3 binding, as cleaved CD3-engaging formats (DVD-3E C) induced robust STAT5 phosphorylation, whereas the uncleaved form remained largely inactive (Figure 3H). Consistent with these mechanistic findings, treatment of MC38-CLDN tumor-bearing mice with DVD, DVD-IL2, or DVD-3E demonstrated that, despite its attenuated intrinsic activity, DVD-3E retained largely comparable antitumor efficacy to DVD-IL2, while markedly reducing treatment-associated body weight loss (Supplemental Figure 3, J–L). These results show that CD3 engagement provides a critical second input to unlock 3E signaling, establishing a protease-activated, logic-gated T cell activation system that spatially couples TCR stimulation with conditional cytokine signaling.

### DVD-3E enhances T cell activation and sustains antitumor cytotoxicity.

To evaluate the effect of DVD-3E on T cell activity, we conducted in vitro cytotoxicity and metabolic assays using cocultures of human PBMCs with CLDN-expressing SNU1 tumor cells. In this vitro system, we used TCE-3E to mimic the cleaved, tumor-activated form of DVD-3E. After coculture, TCE-3E induced substantially stronger tumor cell killing compared with control proteins (Figure 4A) and also promoted robust CD8 T (Figure 4B and Supplemental Figure 4A) and CD4 T cell proliferation (Supplemental Figure 4, B and C). Moreover, the addition of 3E enhanced downstream T cell signaling, as evidenced by increased phosphorylation of ERK in the MAPK pathway (Figure 4C). Metabolic analysis further revealed an elevation in the oxygen consumption rate of T cells, indicating heightened mitochondrial activity and activation (Figure 4D). In a multiround killing assay, TCE-3E demonstrated superior and sustained tumor killing efficacy (Figure 4E). To assess the antitumor activity of DVD-3E in vivo, we established an MC38-CLDN tumor model in hCD3EDG-KI mice. Mice were treated with equimolar doses of TCE or DVD-3E. DVD-3E induced marked tumor regression (Figure 4F) along with reduced peripheral toxicity (Figure 4G and Supplemental Figure 4, D–F). Similarly, in the B16-CLDN tumor model, DVD-3E exhibited superior antitumor efficacy (Figure 4H). Additionally, DVD-3E efficacy was further potentiated by combination with cisplatin (Figure 4I), which demonstrated potent cytotoxicity against both high and low antigen-expressing tumor cells (Supplemental Figure 4G), representing a promising strategy to overcome tumor heterogeneity (Supplemental Figure 4H). To further evaluate the versatility of the DVD platform, we replaced the CLDN-targeting domain with an MSLN-specific binding module while preserving the remaining molecular architecture. The resulting MSLN-directed DVD-3E demonstrated durable antitumor activity in MSLN^+^ tumor models, suggesting that the DVD-3E framework can be readily adapted to target diverse TAAs (Supplemental Figure 4I). Taken together, these results demonstrate that the DVD-3E design achieves enhanced antitumor activity while minimizing systemic toxicity.

### DVD-3E relies on both CD4^+^ and CD8^+^ T cells and requires IFN-γ–mediated tumor control.

To further determine which T cell subsets play a decisive role in DVD-3E–mediated tumor control, we performed in vivo depletion experiments. Depletion of neither CD4^+^ nor CD8^+^ T cells impaired the therapeutic efficacy of DVD-3E (Figure 5A). Complete loss of tumor control was observed only when both CD4^+^ and CD8^+^ T cells were simultaneously depleted (Figure 5B). Together, the data indicate that either subset is functionally able to mediate antitumor activity. The contribution of CD8^+^ T cells residing in tumor-draining lymph nodes (TDLNs) versus the TME during treatment remains a matter of debate (23, 24). To determine whether preexisting cytotoxic T lymphocytes are sufficient for tumor control following DVD-3E treatment or if continuous recruitment of new T cells from lymphoid tissues is required, we administered FTY720 to block lymphocyte egress from TDLNs into the tumor (25). FTY720 treatment efficiently and rapidly depleted circulating T cells to undetectable levels within PBMCs but did not compromise the therapeutic efficacy of DVD-3E (Figure 5C). These findings suggest that preexisting intratumoral T cells are sufficient to mediate the antitumor activity of DVD-3E. Furthermore, the therapeutic effect of DVD-3E was completely lost upon IFN-γ blockade (Figure 5D), indicating that IFN-γ signaling is essential for DVD-3E–induced tumor regression.

### DVD-3E enhances effector function while preserving stem-like T cell states.

To further investigate the immunophenotypic changes induced by DVD-3E therapy, we analyzed the frequency and composition of TILs following antibody treatment. Immunohistochemical analysis revealed substantially increased CD8^+^ T cell infiltration in tumors from DVD-3E–treated mice compared with control groups (Figure 6A). Consistent with these findings, flow cytometry analysis demonstrated a substantial expansion of intratumoral CD90.2^+^ T cells following DVD-3E treatment compared with conventional TCE treatment (Figure 6B and Supplemental Figure 5A), accompanied by increased frequencies of both CD8^+^ (Figure 6C and Supplemental Figure 5B) and CD4^+^ cells (Supplemental Figure 5D). Functionally, DVD-3E treatment enhanced T cell effector activity, as evidenced by increased frequencies of IFN-γ^+^ cells among both CD8^+^ (Figure 6D and Supplemental Figure 5C) and CD4^+^ cells (Supplemental Figure 5E). Notably, DVD-3E reshaped the differentiation state of intratumoral CD8^+^ T cells by reducing the proportion of exhausted PD-1^+^TIM-3^+^ populations and expanding PD-1^–^TIM-3^–^ subsets (Figure 6E). Similarly, the proportion of TOX^+^ cells was decreased (Figure 6F and Supplemental Figure 5F), whereas tumor antigen–specific T cells were substantially enriched in the DVD-3E–treated group (Figure 6G and Supplemental Figure 5G). DVD-3E treatment also promoted T cell proliferation, as evidenced by increased Ki67 expression (Figure 6H and Supplemental Figure 5H). Collectively, these results indicate that DVD-3E reduces terminal T cell exhaustion while enhancing effector function.

Unexpectedly, in contrast to conventional TCEs, which often drive T cell differentiation toward terminal effector states, DVD-3E treatment resulted in a marked increase in the frequency of TCF1^+^ stem-like T cells (Figure 6I), particularly within the PD-1 low subset (Supplemental Figure 5I). A similar elevation in TCF1^+^ cells was also detected among CD4^+^ T cells (Supplemental Figure 5J). Together, these findings demonstrate that, compared with conventional TCEs, DVD-3E not only augments T cell effector responses and mitigates exhaustion but also preserves the stem-like properties of intratumoral T cells.

### DVD-3E treatment induces systemic antitumor immunity mediated by lymph node memory T cells.

To determine whether the immune response elicited by DVD-3E treatment in the primary tumor could generate systemic antitumor immunity and confer protection against distal lesions, we employed a bilateral tumor model. CLDN^+^ cells were inoculated into the right flank on day 0, followed by CLDN^–^ cells on the left flank on day 8, and mice were treated with equimolar doses of TCE or DVD-3E (Figure 7A). While conventional TCE therapy failed to control local CLDN^+^ tumor growth, DVD-3E treatment led to tumor suppression (Figure 7B). Notably, mice treated with DVD-3E exhibited complete control of distal CLDN^–^ tumor growth, whereas TCE induced only transient tumor inhibition (Figure 7C).

Previous studies have shown that systemic tumor control often relies on draining lymph nodes (26–28). Phenotypic analysis of TDLNs revealed that DVD-3E treatment markedly increased the total CD8^+^ T cell population (Figure 6A) and markedly expanded the frequency of antigen-specific tetramer^+^CD8^+^ T cells (Figure 7D) compared with TCE treatment. These antigen-specific T cells predominantly exhibited a CD44^+^CD62L^+^ phenotype (Figure 7E), consistent with a central memory T cell profile known to mediate long-term immune protection (29). In addition, we observed a substantial increase in the naive-like CD44^–^CD62L^+^ population (Figure 6B), whereas the terminal effector CD44^+^CD62L^–^ subset was markedly reduced in the DVD-3E group (Supplemental Figure 6C). Moreover, DVD-3E treatment elevated the proportion of CD103^+^TCF1^+^CD8^+^ T cells within TDLNs (Supplemental Figure 6D), suggesting potential migration from the tumor site to the lymph nodes (30). Simultaneously, a higher frequency of Ki67^+^TCF1^+^ T cells was detected (Figure 6E), indicating enhanced proliferative and self-renewal capacity of these cells. To further evaluate the functional relevance of these TDLN-derived T cells, we transferred lymph node cells from DVD-3E– or TCE-treated donor mice into naive recipients (Figure 7F). Remarkably, adoptive transfer of DVD-3E–primed lymph node cells resulted in complete tumor eradication in all recipient mice, whereas TCE-derived cells failed to control tumor progression (Figure 7G). Consistent with these findings, mice that had completely rejected MC38-CLDN tumors following DVD-3E therapy exhibited durable immune protection upon rechallenge with either MC38 (Figure 7H) or MC38-CLDN (Figure 7I) tumor cells, both of which failed to establish growth. These results collectively demonstrate that DVD-3E therapy not only induces robust local tumor regression but also promotes durable, systemic antitumor immunity mediated by long-lived memory T cells. The data also suggest that T cells for systemic protection might be derived from the TME.

## Discussion

Our findings establish a mechanistic framework through which spatially confined activation of new TCEs can elicit much more potent local and durable systemic antitumor immunity than current TCE. By integrating a protease-activatable DVD, Pro-TCE, with a tumor-dependent IL-2 signaling module, DVD-3E achieves precise control over T cell activation within the TME. This design not only enhances the cytotoxic and proliferative capacity of intratumoral T cells but also preserves their stem-like potential, as reflected by the expansion of TCF1^+^ progenitor and central memory subsets. Notably, the generation of these long-lived memory-like populations in the draining lymph nodes underlies the capacity of DVD-3E to mediate protection against antigen^–^ distal tumors and to confer durable immune memory upon rechallenge.

The rationale for employing tumor-associated protease activity as an activation mechanism is supported by extensive evidence demonstrating that aberrant proteolysis represents a conserved feature of the TME (31). Although the magnitude and composition of protease activity vary among tumor types, multiple classes of proteases, particularly MMPs, are frequently enriched in solid tumors. Both membrane-associated and soluble MMPs have been implicated in tumor-associated proteolysis, providing an attractive biochemical signature for selective drug activation (32). Consistent with this concept, previous studies have demonstrated that tumor-associated MMP activity can be exploited as an endogenous trigger for selective activation of therapeutic molecules (33). In our study, MMP inhibition substantially reduced the antitumor activity of DVD, supporting a functional contribution of MMP-mediated proteolysis to DVD activation. We acknowledge that soluble proteases may also participate in DVD cleavage. However, the tumor-targeting arm of DVD facilitates preferential accumulation and prolonged retention within the TME, where local protease activity and substrate exposure are increased relative to peripheral tissues. Thus, spatial enrichment of DVD within tumors, together with elevated local proteolytic activity, is expected to favor tumor-restricted activation. Future systematic profiling of protease activity landscapes across tumor types will be valuable for identifying tumor contexts most suitable for protease-responsive immunotherapies and guiding clinical translation.

Distinct from current Pro-TCE strategies that prioritize toxicity reduction at the expense of efficacy (8, 34, 35), the DVD design offers a more balanced and mechanistically tunable control. Traditional Pro-TCEs rely on high-affinity masking peptides that often rebind after proteolytic cleavage, thereby limiting full activation and requiring supraphysiologic doses to achieve therapeutic efficacy (36). In contrast, the DVD format employs a steric interface that partially attenuates CD3 engagement sufficient to prevent systemic activation, yet permissive for irreversible unmasking within the protease-rich TME. This design achieves a graded activation effect: despite only moderate attenuation in vitro, DVD-3E exhibited a profound reduction of in vivo toxicity, indicating that complete masking is not a prerequisite for safety and that mild, non-rebinding masks may define an optimal therapeutic window.

Current IL-2 engineering strategies have aimed to selectively bias receptor usage by modulating affinities toward IL-2Rα and IL-2Rβγ complexes (19). Variants have been developed that (a) reduce IL-2Rα binding to minimize Treg expansion (37, 38), (b) enhance IL-2Rβ engagement to potentiate effector T cell activity (39–41), and (c) simultaneously modulate both interactions to fine-tune tissue distribution and safety (42, 43). The 3E variant used in this study exhibits reduced affinity for both IL-2Rα and IL-2Rβ, a design initially expected to limit TCF1^+^ progenitor maintenance. Unexpectedly, we observed a substantial expansion of TCF1^+^ populations within the TME and TDLN, an effect absent in our other IL-2–based therapeutics. This finding raises the intriguing possibility that IL-2Rβγ, rather than IL-2Rα, signaling may play a dominant role in sustaining TCF1 expression and stem-like potential. While α-biased IL-2 variants such as IBI363 may further refine the therapeutic index by restricting activation to CD25^hi^ tumor-resident CD8^+^ T cells and peripheral Tregs (44), our data suggest that βγ-driven signaling may also be harnessed to preserve T cell fitness and memory differentiation.

A recent report using IL-2–armored pMHC bispecific antibodies (45) demonstrated that cytokine fusion can augment antitumor activity with manageable safety. However, these types of designed TCEs have limited efficacy; more importantly, they fail to target pan-CD3^+^ T cells, the most potent yet toxicity-prone effector population. Similarly, the local delivery of oncolytic adenovirus vectors to codeliver TCEs and IL-2 circumvents (46), but does not solve, the molecular safety engineering needed for protein-based, systemically deliverable TCEs, which remain the clinical standard.

Conventional TCEs often drive precursor or bystander T cells into terminally differentiated cytotoxic states, leading to activation-induced cell death and exhaustion. In contrast, DVD-3E replenishes and sustains the TCF1^+^ progenitor-like CD8^+^ T cell pool through its integrated CD3 and IL-2 signaling, while simultaneously reducing expression of exhaustion markers such as Tim-3 and TOX. These effects likely underlie its capacity to maintain a regenerative, self-renewing T cell compartment within tumors. Moreover, given that IL-15 shares IL-2Rβγ signaling components and exerts stronger proliferative effects (47), future studies may explore IL-15–based modules to further enhance the persistence and proliferative fitness of tumor-reactive T cells in TCE platforms.

Collectively, these findings demonstrate that engineering spatially restricted cytokine signaling into conditionally active TCEs can overcome both the safety and durability barriers of conventional TCEs. DVD-3E exemplifies a next-generation logic-gated platform that integrates protease-dependent activation with finely tuned IL-2 signaling to achieve immune regeneration and long-term antitumor protection.

Several limitations of this study should be considered. Although DVD-3E demonstrated antitumor activity in multiple preclinical tumor models, further evaluation across a broader range of tumor types and validation in more clinically relevant systems, including patient-derived organoid models and human tumor tissue–based platforms, is important to better define its translational potential and optimal clinical applications. In addition, although DVD-3E effectively restored IL-2 signaling and enhanced T cell activation, additional immunosuppressive mechanisms within the TME, including the accumulation of immunosuppressive cell populations and metabolic constraints, may limit therapeutic responses. Future studies combining DVD-3E with approaches that enhance endogenous antitumor immune priming, such as tumor vaccination, or target complementary immunosuppressive pathways may further improve therapeutic efficacy and broaden its clinical applicability.

## Methods

### Sex as a biological variable.

The study utilized 8- to 10-week-old male and female mice. Sex was not considered as a biological variable in this study. Individual experiments were conducted using mice of the same sex, and no direct comparisons between male and female mice were performed.

### Mice.

C57BL/6 and RAG1-KO mice were purchased from Gempharmatech. hCD3EDG mice (catalog NM-HU-220120) were purchased from Shanghai Model Organisms Center. The mice were randomized and assigned to experimental groups at the start of the experiment. Mice were kept at 12 h light/12 h dark cycles with unrestricted food and water availability.

### Cell lines.

SNU1, L293, MC38, and B16 cells were obtained from the American Type Culture Collection. HEK-Blue IL-2/IL-15 cells were purchased from InvivoGen and Expi293F cells from Thermo Fisher Scientific. The SNU1-hCLDN, MC38-mCLDN, and B16-mCLDN cell lines were generated by lentiviral overexpression of CLDN, followed by single-cell cloning via limiting dilution. MC38/MC38-mCLDN, B16/B16-mCLDN, and HEK-Blue IL-2/IL-15 cells were cultured in DMEM (Gibco) supplemented with 10% FBS (ExCell Bio), 1% penicillin-streptomycin (Gibco), 1% nonessential amino acids (Gibco), 1% sodium pyruvate (Gibco), and 25 mM HEPES (Gibco). SNU1/SNU1-hCLDN cells and human PBMCs were cultured in RPMI-1640 medium (Gibco) supplemented with the same additives. Expi293 cells were maintained in SMM293-T II expression medium (Sino Biological, M293TII). All cells were incubated at 37°C in an atmosphere containing 5% CO_2_.

### Antibody production and design.

Antibodies were generated using the heterodimeric Fc variant technology KiHss-AkKh, as previously described (48). The anti-CLDN fragment was fused to the knob variant Fc region and the anti-CD3 (UCHT1) fragment to the hole variant Fc region. Antibodies were expressed by transient cotransfection in Expi293F cells, harvested 7 days after transfection, and purified by protein A affinity chromatography on an ÄKTA system. Purified antibodies were neutralized with Tris-HCl, sterile filtered (0.22 μm), and analyzed for purity and heterogeneity by SDS-PAGE and HPLC.

To abrogate Fc effector function, LALA-PG mutations were introduced into the CH2 domain. A DVD format was employed, incorporating a cleavable linker (SGWLPSSITA) between the CLDN and CD3 variable domains. IL-2, fused to the C-terminus of the anti-CLDN Fc chain via a G4S linker, carried 3 mutations (K35E, R38E, and K43E) to generate the 3E variant.

### Cell-based binding assay.

Target protein–expressing cells were prepared as single-cell suspensions (2 × 10^6^ cells/mL in PBS) and seeded into V-bottom 96-well plates (100–200 μL/well). Serial 10-fold dilutions of test proteins (6 concentrations) were incubated with cells for 30 min on ice. After washing with FACS buffer (PBS with 2% FBS, 1 μM EDTA, and 0.1% NaN_3_), cells were stained with PE-conjugated anti-human IgG antibody (0.3%) for 30 min, washed, resuspended in FACS buffer, and analyzed by flow cytometry. Naive human PBMCs were used for CD3 binding, MC38-CLDN cells for CLDN binding, and HEK-IL2 or L293 cells overexpressing IL-2Rα, IL-2Rβ, or IL-2Rγ for IL-2 binding.

### In vitro T cell activation and exhaustion assay.

T cells were stimulated with plate-bound anti-CD3 (OKT3, 1 μg/mL) and anti-CD28 (CD28.2, 5 μg/mL) in RPMI-1640 medium (2 × 10^6^ cells/mL) for 2 days at 37°C with 5% CO_2_, and activation was assessed by CD25 and CD69 coexpression. To induce exhaustion, cells were restimulated every 2 days on freshly coated plates for 4–5 cycles, after which exhaustion was evaluated by PD-1 and Tim-3 coexpression.

### In vitro cytotoxicity assay.

Human PBMCs were used as effector cells and cocultured with tumor target cells (SNU1/SNU1-hCLDN) at defined effector/target ratios (1:4 for single-round and 1:3 for multiround assays) in U-bottom 96-well plates. Cells were treated with equimolar TCE-3E or control proteins (20 nM) and incubated for 36–72 h. After incubation, cells were lysed and bioluminescence was measured at 560 nm using a luciferase reporter assay (YESEN, Luciferase Reporter Gene Assay Kit, 11401ES60) to quantify viable cells. Percent target cell lysis was calculated relative to untreated controls. For multiround killing, fresh target cells were added every 48 h to maintain the effector/target ratio, and cumulative cytotoxicity was assessed as above.

### MMP cleavage of fusion protein.

Recombinant human MMP14 (MT1-MMP, R&D Systems) was activated using 0.1 μg/mL rhFurin in activation buffer, which comprises 50 mM Tris, 0.15 M NaCl_2_, 10 mM CaCl_2_, 5 μM ZnCl_2_, and 0.05% Brij-35, pH 7.5, incubated at 37°C for 2 h. Activated MMP14 was used to cleave 1 μg fusion protein in assay buffer (50 mM Tris, 3 mM CaCl_2_, and 5 μM ZnCl_2_) for 2 h at 37°C. Cleavage was confirmed by SDS-PAGE.

### Analysis of pSTAT5 in T cells.

IL-2–induced STAT5 phosphorylation was assessed in activated human PBMCs. Equal molar DVD-3E and control proteins were prepared at 20 and 2 nM. Activated T cells were rested for 4 h in serum-free RPMI-1640, then 1 × 10^6^ cells were plated in V-bottom 96-well plates with or without SNU1-hCLDN. After centrifugation and removal of the supernatant, cells were resuspended with soluble proteins and incubated for 25 min at 37°C. Cells were fixed with 4% paraformaldehyde, permeabilized with methanol, and stained for pSTAT5 for flow cytometry analysis.

### Reporter measurements in HEK-Blue IL-2/IL-15 cells.

The activity of fusion proteins with different IL-2 positions was evaluated using HEK-Blue IL-2/IL-15 reporter cells. Cells (2.8 × 10^4^ per well) were plated in flat-bottom 96-well plates and incubated with antibodies for 24 h at 37°C. Supernatants were mixed with QUANTI-Blue solution (InvivoGen) and incubated for 1–3 h at 37°C. Secreted embryonic alkaline phosphatase (SEAP) levels were quantified spectrophotometrically at 620–655 nm.

### Bulk RNA-seq.

Exhausted and freshly activated T cells were washed twice with PBS, resuspended in Trizol, and stored at −80°C. Bulk RNA-seq, including data collection, processing, and preliminary analysis, was performed by Annoroad Gene Technology. Data normalization, differential expression analysis, and principal component analysis were conducted using the R package DESeq2 (v1.18.1). Heatmaps were generated using the R package Complex Heatmap (v2.12.0) in RStudio.

### Tumor digestion and homogenate preparation.

Tumor tissues were mechanically homogenized and enzymatically digested using a solution containing 0.5 mg/mL collagenase IV (Sigma-Aldrich) and 1 mg/mL DNase I (Roche) at 37°C for 45 min under gentle agitation. The resulting tumor cell suspension was then passed through a 70 μm cell strainer to eliminate any remaining undigested tissue fragments. Following filtration, the cells were washed twice with PBS supplemented with 2 mM EDTA and 2% fetal calf serum.

### In vivo animal study protocol.

For the syngeneic mouse model, hCD3EDG-KI mice were s.c. injected with 1 × 10^6^ MC38-CLDN or 5 × 10^5^ B16-CLDN cells into the right flank. When tumors reached approximately 100–120 mm³, tumor-bearing mice were randomly assigned to treatment groups. Mice received i.p. injections of about 200 pmol of the fusion proteins.

For the immune deficiency mouse model, RAG1-KO mice (8 weeks old) were i.v. injected with 2 × 10^6^ hCD3EDG-KI mice splenocytes. One day later, they were inoculated s.c. with 1 × 10^6^ MC38-CLDN cells on the right flank. Ten days after splenocyte injection, treatment was initiated. Tumor volumes were measured using calipers and calculated with the formula (length × width × width)/2. Body weights were also recorded.

For cell depletion, 200 μg of depleting antibodies was administered i.p. 1 day before treatment and before each subsequent treatment.

For FTY720 treatment, 1 day before treatment initiation, mice received an i.p. dose of 20 μg FTY720, followed by 10 μg doses every 48 h for 1 week.

For MMP inhibition, 500 μg GM6001(MMP inhibitor, Targetmol) was administered every other day, beginning 2 days prior to the initiation of DVD treatment and continuing throughout the entire treatment period and for an additional 7 days after the last DVD dose.

### Monitoring and sample collection.

Body weights were monitored regularly starting from treatment initiation. Peripheral blood was collected at 24 or 36 h after treatment and centrifuged to isolate serum. Cytokine levels were quantified using cytometric bead array (CBA) kits: the Human Th1/Th2 Cytokine CBA Kit II (550749, BD Biosciences) for the human PBMC–related in vitro system and the Mouse Inflammation CBA Kit (552364, BD Biosciences) for the in vivo system. All procedures were performed according to the manufacturer’s instructions.

### SPR analysis.

SPR measurements were performed using a Biacore T200 instrument (Cytiva). CD3ε was diluted to 10 μg/mL in 10 mM sodium acetate buffer (pH 5.0) and immobilized on the activated sensor surface to approximately 75 RU. Serial dilutions of TCE and DVD proteins were prepared in running buffer (HBSS+, pH 7.4) at 100 nM for kinetic analysis.

### Flow cytometry.

For flow cytometry analysis, cells were resuspended in FACS buffer (PBS without Ca²^+^ and Mg²^+^, Gibco) containing 2% FBS (Gibco) and 1 mM EDTA. Single-cell suspensions from mouse tissues or tumors were incubated for 20 min on ice with mouse Fc block at a dilution of 1:100 (BioLegend, 101302, clone 93) and Fixable Viability Dye eFluor 506 (Thermo Fisher Scientific, 65-0866-18). Cells were then incubated with primary antibodies for 30 min at 4°C in the dark. The following antibodies were used: against mouse CD8 (MBL, K0227-4, clone KT15), anti-mouse TCF1 (BD Biosciences, 566693, clone S33-966), anti-mouse CD44 (BD Biosciences, 103039, clone IM7), anti-mouse CD62L (BioLegend, 104440, clone MEL-14), anti-mouse CD90.2 (Thy-1.2) (BioLegend, 140318, clone 53-2.1), anti-mouse CD279 (PD-1) (BioLegend, 135225, clone 29F.1A12), anti-mouse CD45 (BioLegend, 103106, clone 30-F11), anti-mouse CD103 (BioLegend, 121432, clone 2E7), anti-mouse CD366 (TIM3) (BioLegend, 134010, clone B8.2C12), anti-mouse STAT5(pTyr694) (CST, 9365S, clone C71E5), anti-mouse H-2Kb MuLV p15E tetramer (MBL, TS-M507-1), anti-mouse F4/80 (BioLegend, 123141, clone BM8), anti-mouse CD11b (BioLegend, 101236, clone M1/70), anti-mouse CD8a (BioLegend, 100730, clone 53-6.7), anti-mouse CD132 (common γ chain) (BioLegend, 132308, clone TUGm2), anti-mouseCD279 (PD-1) (BioLegend, 135208, clone 29F.1A12), anti-mouse IFN-γ (BioLegend, 505808, clone XMG1.2), anti-mouse CD25 (BioLegend, 102008, clone PC61), anti-mouse CD69 (BioLegend, 104506, clone H1.2F3), anti-mouse NK1.1 (BioLegend, 108706, clone PK136), anti-human CD45 (BioLegend, 368536, clone 2D1), anti-human CD25 (BioLegend, 302603, clone BC96), anti-human CD8 (BioLegend, 344706, clone SK1), anti-human ERK1/2 phospho(Thr202/Tyr204) (BioLegend, 675503, clone 4B11B69), anti-human CD366(Tim-3) (BioLegend, 345026, clone F38-2E2), and anti-human CD279(PD-1) (BioLegend, 329920, clone EH12.2H7).

### Immunohistochemistry.

Paraffin-embedded sections were routinely deparaffinized, rehydrated, and subjected to antigen retrieval. Endogenous peroxidase activity was blocked using 3% H_2_O_2_, followed by serum blocking. The sections were then incubated overnight at 4°C with the primary antibody against CD8 (Servicebio, GB15068; dilution: 1:500). After washing with PBS, the sections were incubated with an HRP-conjugated goat anti-rabbit IgG secondary antibody (Servicebio) at room temperature for 50 min. Immunoreactivity was visualized using 3,3′-diaminobenzidine, followed by counterstaining with hematoxylin, then mounted for microscopy examination.

### Statistics.

Data are presented as mean ± SEM unless otherwise noted. Statistical analysis for each experiment is specified in the corresponding figure legend. Statistical analyses were performed using GraphPad Prism 8 software. For comparisons between 2 groups, 2-tailed unpaired Student’s *t* test was applied. For multiple group comparisons, 1- or 2-way ANOVA was used as appropriate. *P* values less than 0.05 were considered significant in all cases.

### Study approval.

All mice were maintained under specific pathogen–free conditions in the animal facility of Chinese Institute for Brain Research (CIBR), Beijing, China. All experimental procedures were performed in accordance with and approved by the IACUC of CIBR (protocol CIBR-IACUC-048).

### Data availability.

The data supporting this study are available within the article and its supplemental information. Numerical data underlying the graphs and plots presented in the main and supplemental figures are provided in the Supporting Data Values file. Bulk RNA-seq data generated in this study have been deposited in the NCBI Gene Expression Omnibus under accession number GSE346542. Additional data generated during this study are available from the corresponding author upon reasonable request.

## Manuscript information

### Author contributions.

ZY, YXF, and SL conceived and designed the study. ZY and SL designed the experimental procedures and data analysis and wrote the initial manuscript. SL and NL performed all experiments. ZY, XL, YC, MW, and HW performed experiments and/or data interpretation. ZY and YXF supervised the study. All authors reviewed the manuscript.

### AI disclosure.

The authors did not use AI tools for research design, data extraction, data analysis, data interpretation, or preparation of figures, graphics, or other research content. ChatGPT (OpenAI) was used solely to assist with manuscript English language editing, grammar correction, and polishing during the preparation and revision of this article from July 2025 to September 2026. All AI-assisted text was carefully reviewed and verified by the authors, and the authors take full responsibility for the accuracy and content of the manuscript.

### Conflict of interest.

ZY, YXF, SL, and NL are listed as coinventors on the patent “Bispecific antibody prodrug and its application,” owned by Changping Laboratory. ZY, YXF, SL, and NL are listed as coinventors on the patent “Bispecific antibody and cytokine fusion protein and its application,” owned by Changping Laboratory.

### Funding.

This study was supported by the following:

- Noncommunicable Chronic Diseases-National Science and Technology Major Project (2023ZD0520204).
- Changping Laboratory (2021C-02-04 and 2026C-02-04).

## Supplementary Material

Supplemental data

Unedited blot and gel images

Supporting data values

## Figures and Tables

**Figure 1:**
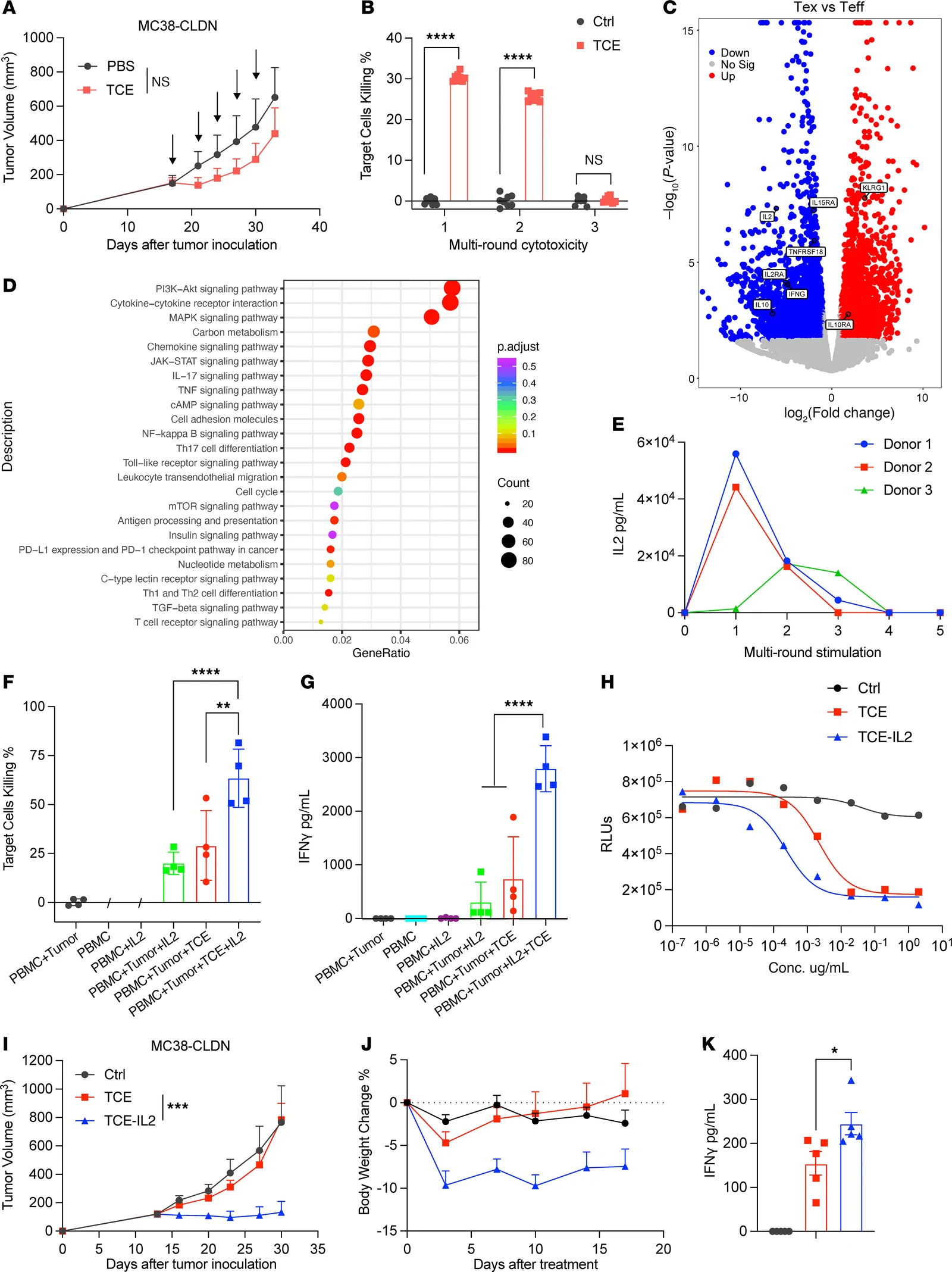
Exogenous IL-2 enhances TCE-mediated T cell cytotoxicity while maintaining effector T cell functionality. (**A**) Growth of MC38-CLDN tumors in hCD3-KI mice. Mice were randomly grouped and i.p. treated with PBS or 200 pmol TCE once every 3 days for 5 doses. *n* = 5 mice per group. (**B**) Multiround killing of SNU1-CLDN cells by TCE in human PBMC coculture, with an effector target ratio of 1:3; tumor cell viability was assessed every 48 h. (**C**) Volcano plot of differentially expressed genes between effector T (Teff) and exhausted T (Tex) cells. Genes with adjusted *P* < 0.01 are shown; upregulated genes are highlighted in red and downregulated genes in blue. (**D**) KEGG pathway enrichment bubble plot based on Tex_Teff downregulated genes. Only pathways with an adjusted *P* value < 0.05 are shown. (**E**) PBMCs from different donors were repeatedly stimulated with human T cell-activating CD3/CD28 Dynabeads. Culture supernatants were collected every 48 h for IL-2 measurement, followed by the addition of fresh beads. (**F** and **G**) Tumor cell killing by human PBMCs (**F**) and IFN-γ levels in culture supernatants or in the presence of TCE, IL-2, or both (**G**), cocultured with an effector-to-target (E/T) cell ratio of 1:3 for 72 h. (**H**) Tumor cell killing by human PBMCs in serial dilutions of TCE or TCE-IL2 with an E/T ratio of 1:1 for 48 h. (**I**–**K**) Tumor growth (**I**) and body weight change (**J**) in the MC38-CLDN model following 200 pmol TCE or TCE-IL2 treatment. Serum cytokine (**K**) levels 24 h after first treatment. *n* = 5 mice per group. Data are shown as mean ± SEM and are representative of at least 2 independent experiments. Individual data points shown in the figures represent independent biological replicates. Statistical analysis was performed using 1-way (**B**, **F**, **G**, and **K**) or 2-way (**A** and **I**) ANOVA. *P < 0.05, **P < 0.01, ***P < 0.001, ****P < 0.0001.

**Figure 2:**
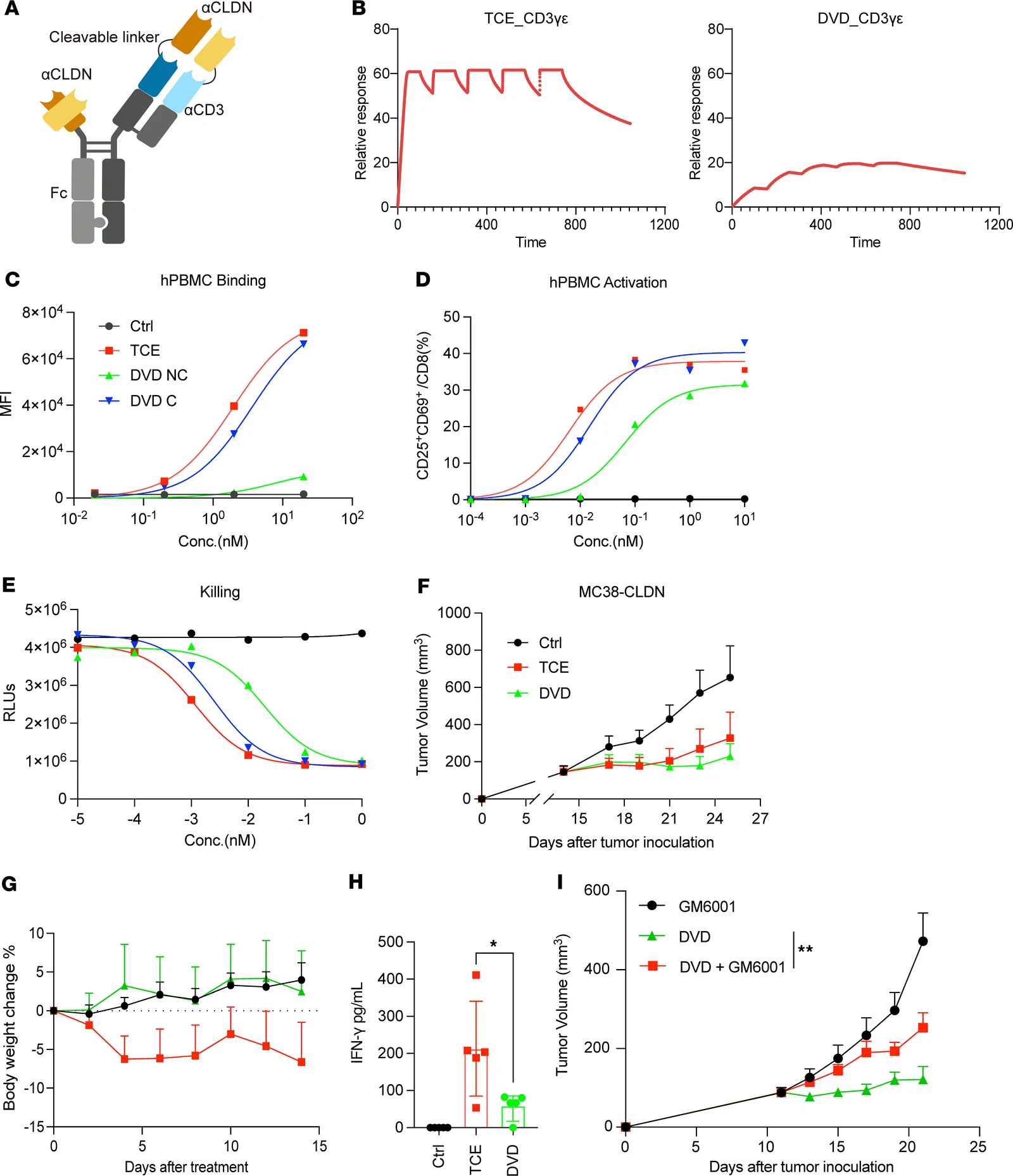
Protease-activatable DVD design suppresses αCD3 activity and restores engagement after cleavage. (**A**) Schematic of the DVD construct. Anti-CLDN variable domains were fused to the N-terminus of the anti-CD3 chain via protease-cleavable linkers. (**B**) Binding affinities of TCE and DVD to hCD3γε measured by SPR. (**C**) TCE, cleaved (C) DVD, or uncleaved (NC) DVD binding of human PBMCs (hPBMC) analyzed by flow cytometry. (**D**) T cell activation (CD25^+^CD69^+^) measured following hPBMC and SNU1-CLDN cocultures with serial dilutions of TCE or cleaved or uncleaved DVD proteins. (**E**) Killing of SNU1-CLDN cells by hPBMCs with E/T ratio of 1:1 for 48 h, treated with PBS, TCE, or DVD. (**F**–**H**) Tumor growth (**F**), body weight changes (**G**), and IFN-γ release (**H**) in the MC38-CLDN–bearing hCD3-KI mice treated with PBS, 200 pmol TCE, or DVD once every 3 days for 6 doses. *n* = 5 mice per group. (**I**) Tumor growth in the MC38-CLDN–bearing hCD3-KI mice treated with or without GM6001 once every 2 days for 5 doses. GM6001 administration was initiated 24 h before DVD-3E treatment. DVD was administered on days 11, 14, and 17. *n* = 5 mice per group. Data are shown as mean ± SEM and are representative of at least 2 independent experiments. Statistical analysis was performed using 1-way (**H**) or 2-way (**I**) ANOVA. *P < 0.05, **P < 0.01.

**Figure 3:**
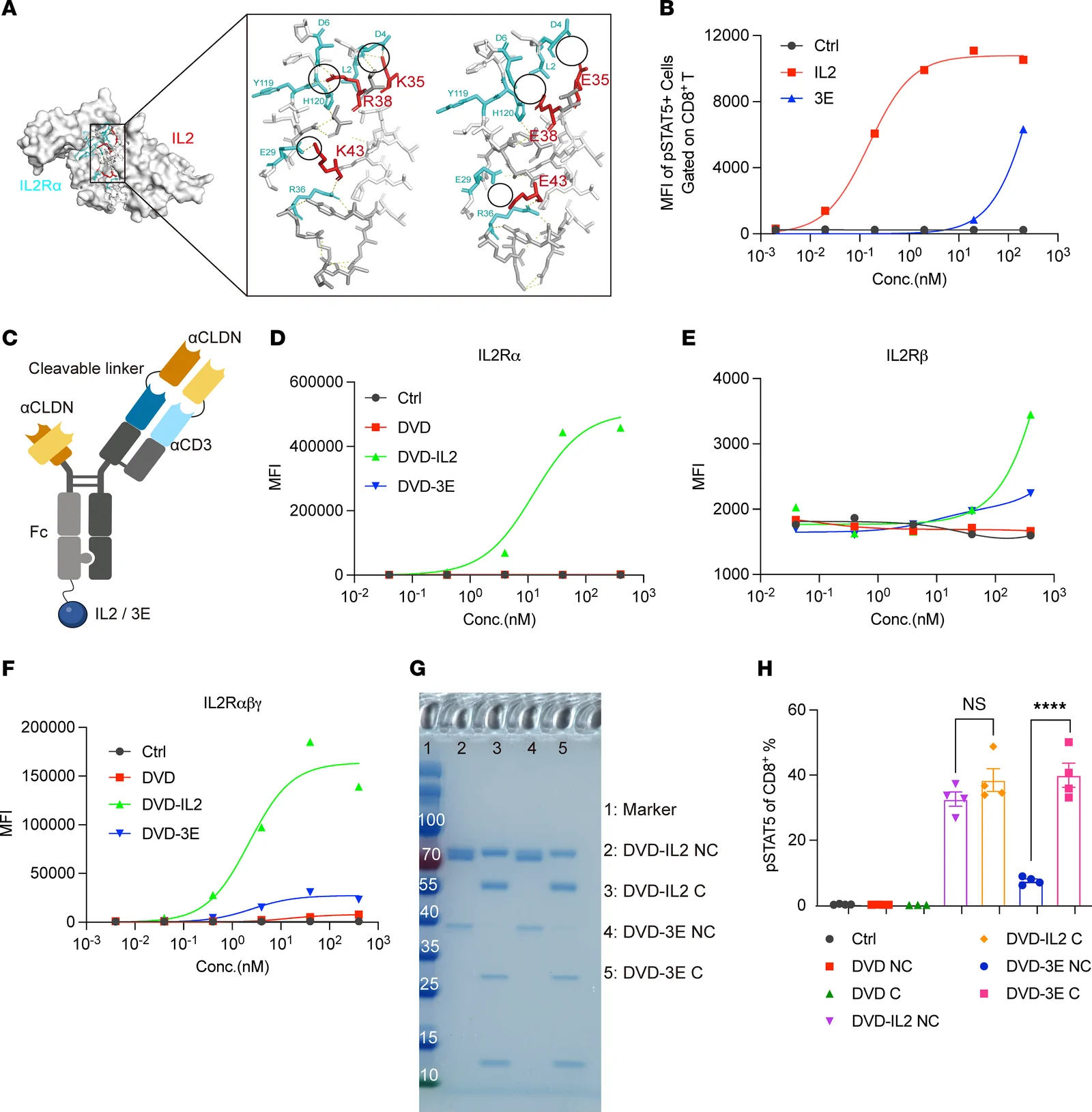
IL2(3E) exhibits reduced receptor binding and αCD3-dependent activity. (**A**) Structural modeling of IL-2 (left) and IL2(3E) (right) binding to IL-2Rα. Key residues of IL2 (red) and corresponding residues of IL2Rα (blue) are shown. (**B**) STAT5 phosphorylation in human PBMCs stimulated with serial dilutions IL-2 or IL2(3E). (**C**) Schematic of the DVD-IL2/3E construct. (**D**–**F**) Binding of IL-2 or IL2(3E) to IL-2Rα (**D**) or IL-2Rβ (**E**) or HEK-Blue IL-2 reporter cells with IL-2Rαβγ (**F**). (**G**) SDS-PAGE analysis of cleaved (C) and uncleaved (NC) DVD, DVD-3E, and DVD-IL2. (**H**) STAT5 phosphorylation in human PBMCs stimulated with cleaved or uncleaved DVD, DVD-IL2, and DVD-3E for 20 min. Data are shown as mean ± SEM and are representative of at least 3 independent experiments. Individual data points shown in the figures represent independent biological replicates. Statistical analysis was performed using 1-way ANOVA (**H**). ****P < 0.0001.

**Figure 4:**
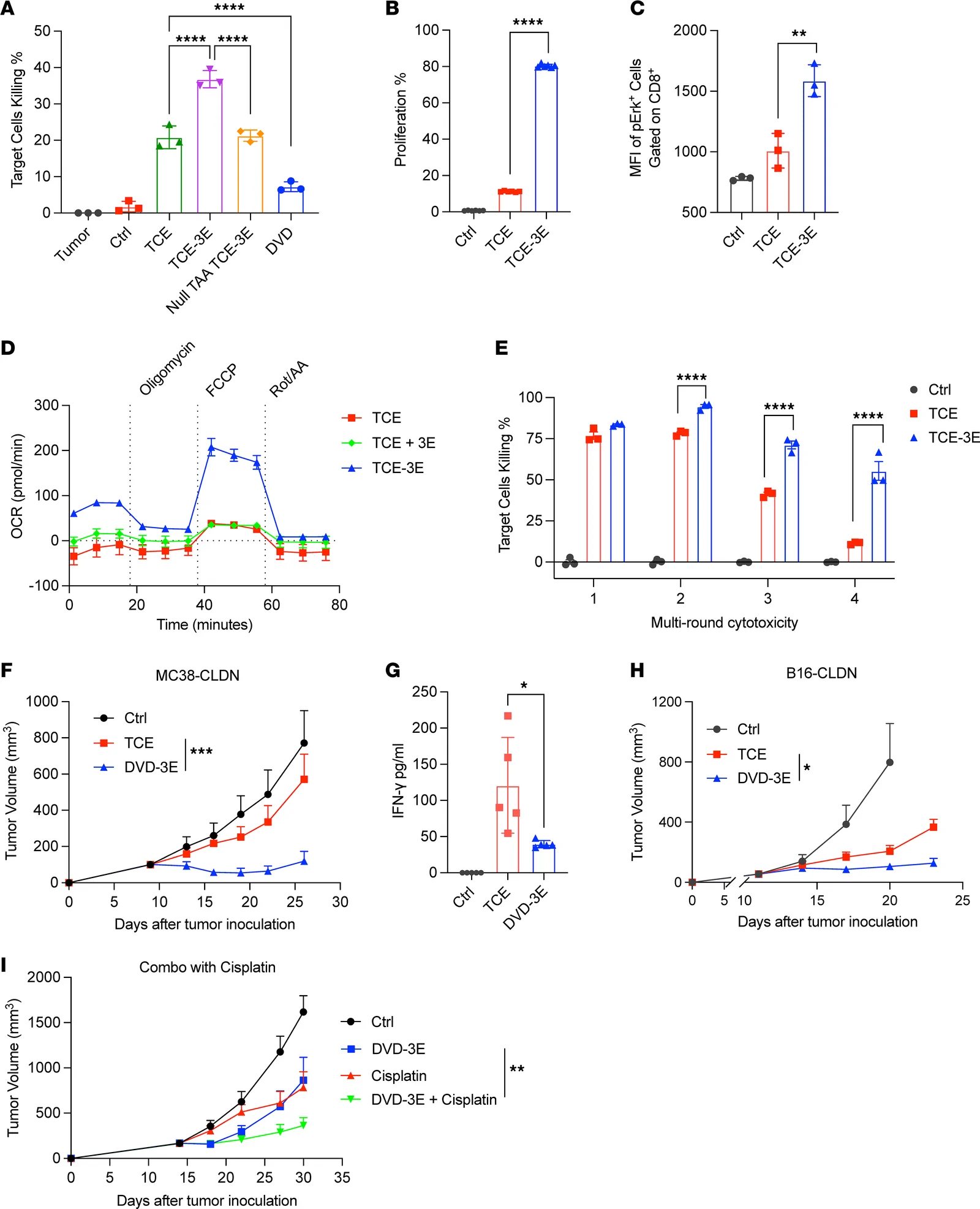
DVD-3E enhances antitumor function without toxicity. (**A** and **B**) Tumor cell killing (**A**) and T cell proliferation (**B**) in human PBMC and SNU1-CLDN cocultures with proteins at a concentration of 20 nmol and assayed after 72 h. (**C**) ERK phosphorylation in human PBMCs cocultured with SUN1-CLDN (E/T = 2:1) and stimulated with TCE or TCE-IL2 for 25 min. (**D**) Oxygen consumption rate (OCR) of activated T cells after treatment with TCE, TCE-3E, or TCE mix with IL2(3E). FCCP, carbonyl cyanide-4 (trifluoromethoxy)phenylhydrazone; Rot/AA, rotenone and antimycin A. (**E**) Multiround killing of SNU1-CLDN cells by Tex cells cocultured with 20 nmol TCE or TCE-3E. (**F**–**H**) Tumor growth (**F**) and IFN-γ release (**G**) in the MC38-CLDN and B16-CLDN (**H**) model treated with PBS, 200 pmol TCE, or DVD-3E once every 3 days for 2 doses. *n* = 5 mice per group. (**I**) Tumor growth in the MC38-CLDN model treated with PBS or 200 pmol DVD-3E. PBS or DVD-3E with or without cisplatin (25 μg on days 15–17 and 50 μg on days 18–20). *n* = 5 mice per group. Data are shown as mean ± SEM and are representative of at least 2 independent experiments. Individual data points shown in the figures represent independent biological replicates. Statistical analysis was performed using 1-way (**A**–**C** and **G**) or 2-way (**E**, **F**, **H**, and **I**) ANOVA. *P < 0.05, **P < 0.01, ***P < 0.001, ****P < 0.0001.

**Figure 5:**
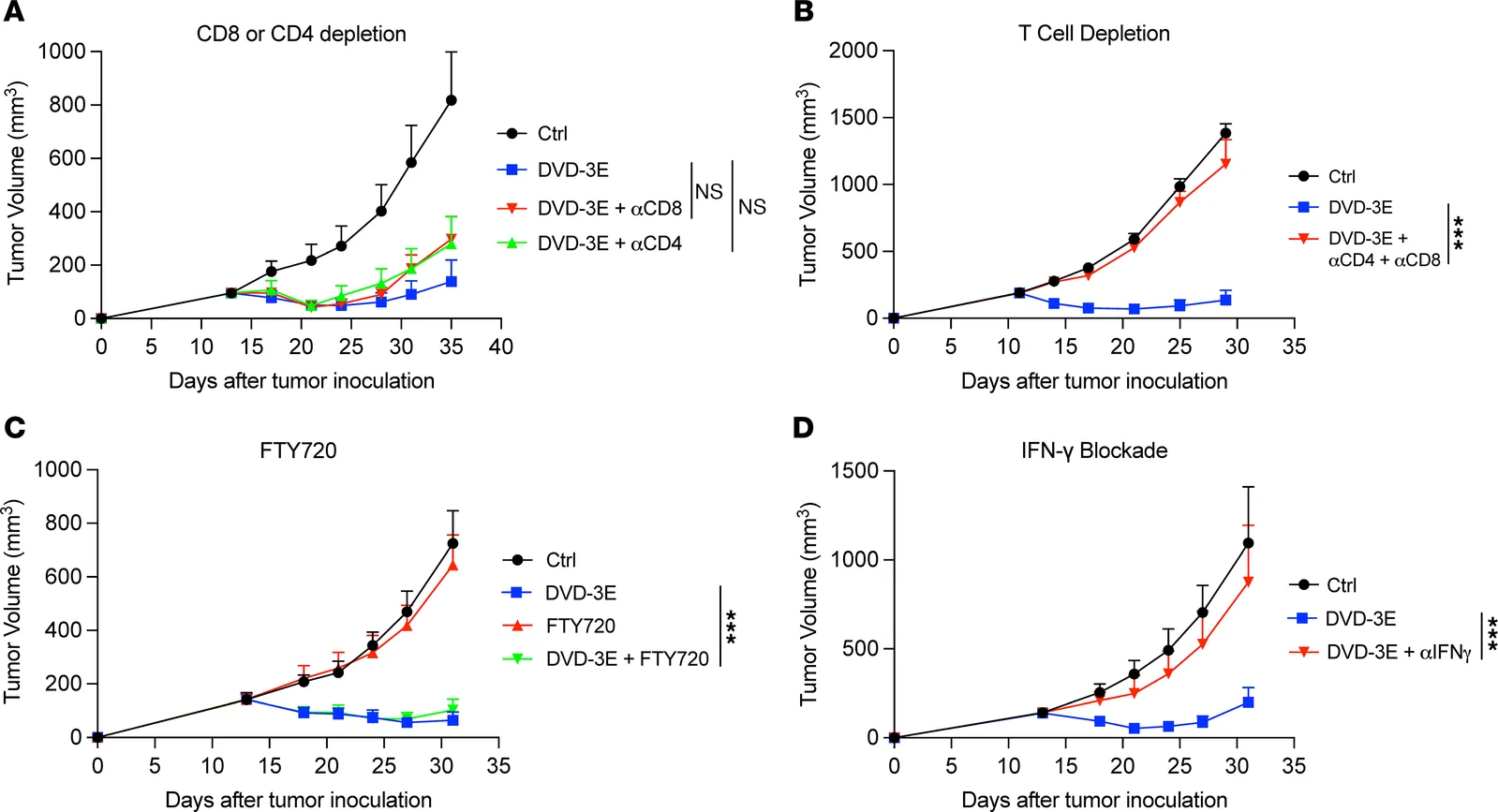
Cellular requirements and therapeutic synergy of DVD-3E in vivo. Growth of MC38-CLDN tumors in hCD3-KI mice treated with PBS or 200 pmol DVD-3E, with or without antibody-mediated depletion of CD4^+^ or CD8^+^ (**A**), CD4^+^ and CD8^+^ (**B**), IFN-γ (**C**), or FTY720 blockade of lymphocyte egress (**D**). *n* = 5 mice per group. Data are shown as mean ± SEM and are representative of at least 2 independent experiments. Statistical analysis was performed using 2-way ANOVA (**A**–**D**). ***P < 0.001.

**Figure 6:**
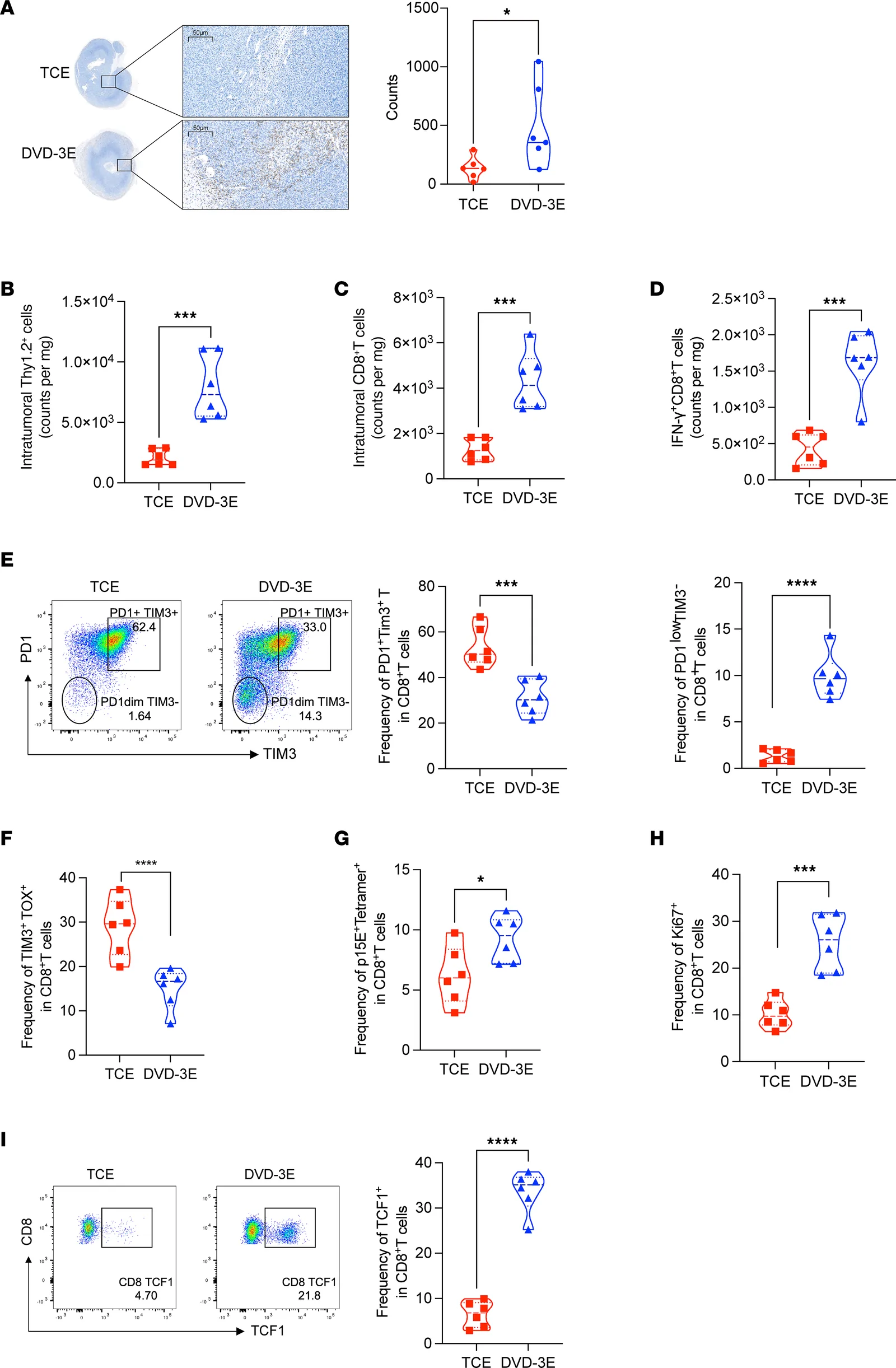
DVD-3E promotes intratumoral T cell proliferation and sustains stem-like CD8^+^ T cells. hCD3-KI mice bearing MC38-CLDN tumors were i.p. treated with 200 pmol TCE or DVD-3E on days 14 and 17. Tumors were harvested 24 h after the second treatment, and TILs were analyzed. (**A**) Representative images of CD8 immunohistochemical staining in tumor sections from the indicated groups and quantification of CD8^+^ T cell numbers per field of view in individual mice. Scale bars: 50 μm. (**B**–**I**) Flow cytometric quantification includes total intratumoral T cells (**B**), CD8^+^ T cells (**C**), IFN-γ^+^CD8^+^ T cells (**D**), PD-1^+^Tim-3^+^CD8^+^ T cells or PD-1^lo^Tim-3^–^CD8^+^ T cells (**E**), TOX^+^CD8^+^ T cells (**F**), p15E^+^tetramer^+^CD8^+^ T cells (**G**), Ki67^+^CD8^+^ T cells (**H**), and TCF1^+^CD8^+^ T cells (**I**). For intracellular cytokine staining, TILs were stimulated ex vivo with PMA and ionomycin. *n* = 6 mice per group. Data are shown as mean ± SEM and are representative of at least 2 independent experiments. Each dot represents an individual mouse. Statistical analysis was performed using 2-tailed unpaired Student’s *t* test (**A**–**I**). *P < 0.05, ***P < 0.001, ****P < 0.0001.

**Figure 7:**
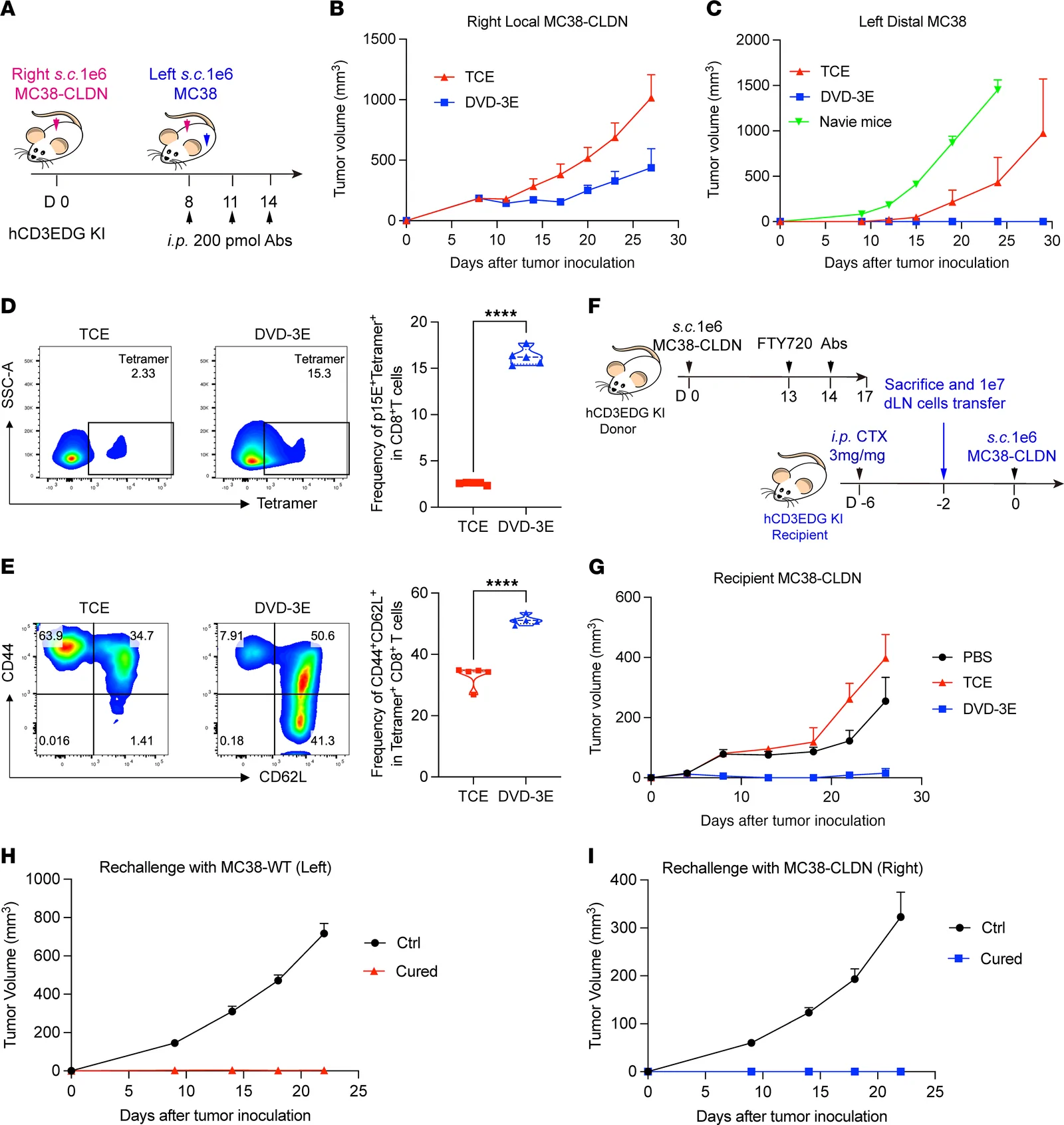
DVD-3E enhances distal and memory immunity in draining lymph nodes. (**A**) Schematic of the bilateral tumor model showing the inoculation and treatment setup. (**B** and **C**) Growth curves of the local (right flank; **B**) and distal (left flank; **C**) tumors. PBS, 50 pmol TCE, and DVD-3E were administered via intratumoral injection once every 3 days for 2 doses. *n* = 5 mice per group. (**D** and **E**) Frequencies of tetramer^+^CD8^+^ T cells (**D**) and CD44^+^CD62L^+^ memory subsets (**E**) in draining lymph nodes. (**F** and **G**) Adoptive transfer experiment schematic (**F**) and tumor growth in recipients (**G**). *n* = 5 mice per group. (**F**) CTX, cyclophosphamide. (**H** and **I**) Tumor rechallenge in cured mice with MC38 (**H**) or MC38-CLDN (**I**). *n* = 5 mice per group. Data are shown as mean ± SEM and are representative of at least 2 independent experiments. Each dot represents an individual mouse. Statistical analysis was performed using 2-tailed unpaired Student’s *t* test (**D** and **E**). ****P < 0.0001.

## Version 1 11/16/2026

Electronic publication
