# Supplementary material for: Logic-gated T cell engager with mutant IL-2 potentiates antitumor responses without cytotoxicity in solid tumor models
Source: J Clin Invest. 2026 Nov 16;136(22):e204657. doi: 10.1172/JCI204657 (PMC13767619; doi:10.1172/JCI204657)
Supplement: Supplementary file 1 [file jci-136-204657-s022.pdf]

Logic-gated T cell engager with mutant IL-2 potentiates anti-tumor responses without cytotoxicity in solid tumor models

Shijie Li[1,2,3,4,5,#], Nan Li[1,#], Xiangming Liu[1,2], Yueqi Cai[1], Mukai Wang[1], Huimin Wang[1,2], Yang-Xin Fu[1,2,3,*], Zaopeng Yang[1,*]

[1]Changping Laboratory, Beijing 102206, China.
[2]Center for Cancer Biology, School of Basic Medical Sciences, Tsinghua University, Beijing 100084, China.
[3]State Key Laboratory of Molecular oncology, Tsinghua University, Beijing 100084, China.
[4]Institute for Molecular and Cellular Therapeutics, Chinese Institutes for Medical Research, Beijing 100069, China.
[5]School of Basic Medical Sciences, Capital Medical University, Beijing 100069, China.
[#]These authors contributed equally: Shijie Li, Nan Li.
[*]Corresponding author:
Yang-Xin Fu, MD, PhD
Tsinghua University
Haidian District, Beijing 100084, China
+86-010-62791142, yangxinfu@mail.tsinghua.edu.cn

Zaopeng Yang, PhD
Changping Laboratory
No. 19 Life Science Park Rd, Changping District, Beijing 102206, China
+86-18510728606, zaopengyang@cpl.ac.cn

Supplementary Figure and Legend

Supplemental Figure 1

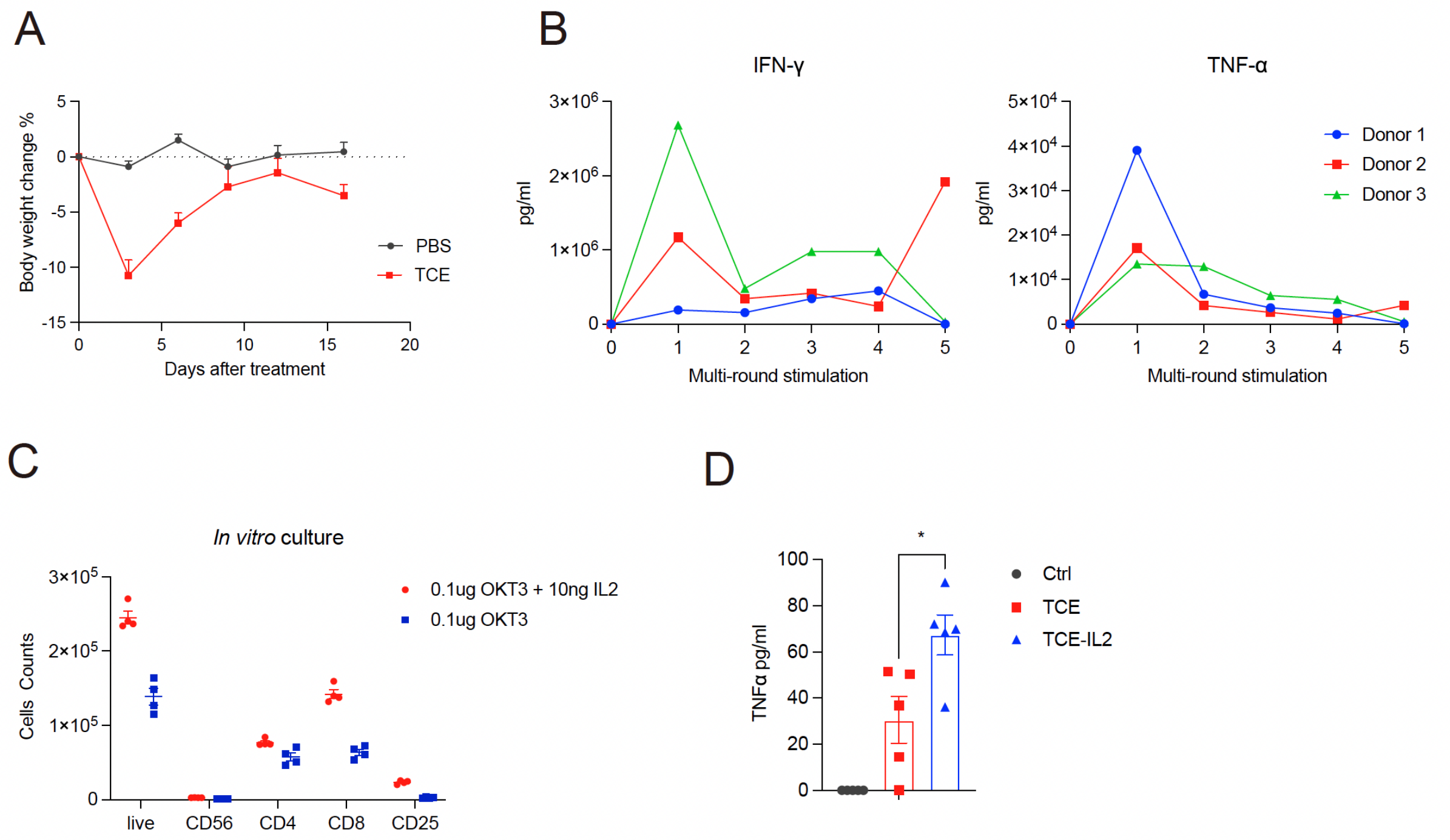

Supplementary Figure 1

A, Bodyweight change of MC38-CLDN tumors in hCD3 knock-in mice treated with PBS or 200 pmol TCE once every 3 days for two doses. n = 5 mice per group.

B, Cytokine secretion profiles of activated and exhausted T cells.

C, huPBMCs were stimulated with anti-CD3 in the presence or absence of IL-2 for 3 days.

D, Serum cytokine levels 24 h after first treatment.in the MC38-CLDN model following 200pmol TCE or TCE-IL2 treatment.

Data are shown as mean ± SEM and are representative of at least two independent experiments. Individual data points shown in the figures represent independent biological replicates. Statistical analysis was performed using one-way ANOVA (**D**) *$P < 0.05$.

Supplemental Figure 2

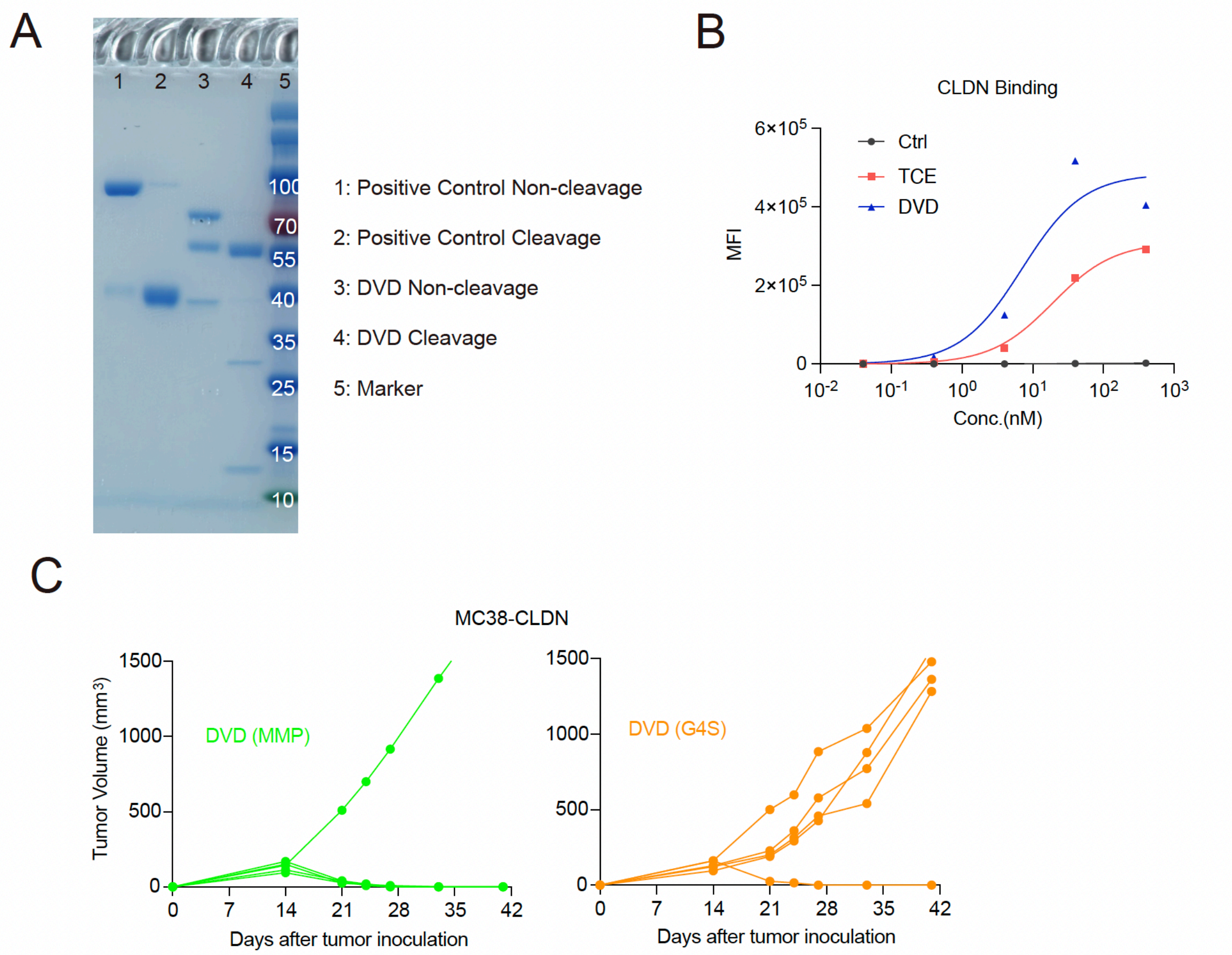

Supplementary Figure 2

A, SDS-PAGE analysis of cleaved and Non-cleaved DVD.

B, Binding affinities of TCE and DVD to CDLN expressed on MC38 cells.

C, Tumor growth curves in RAG1$^{-/-}$ mice reconstituted with 2×10^6 hCD3 knock-in splenocytes. Mice were inoculated with 1×10^6 MC38-CLDN cells and treated intraperitoneally with 100 pmol DVD(G4S) or equimolar DVD on day 14. n = 5 mice per group.

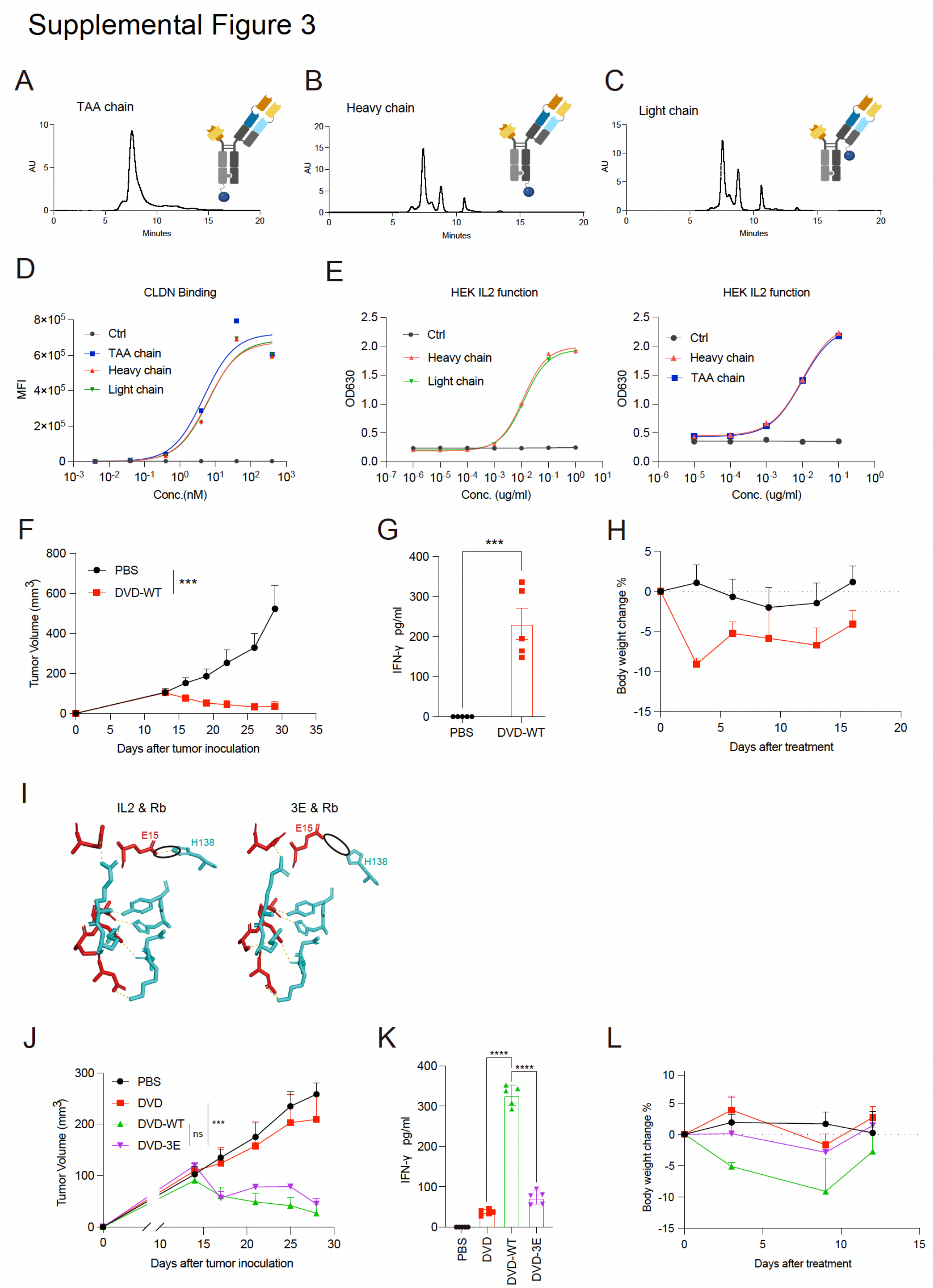

Supplementary Figure 3

A-C, Structural illustration of different fusion protein and HPLC profiles of purified fusion proteins.

D, Binding affinities of different fusion protein to CDLN expressed on MC38 cells.

E, STAT5 phosphorylation in HEK-Blue IL2 reporter cells stimulated with different fusion protein.

F-H, Tumor growth (F), IFN-γ release (G) and body weight (H) in the MC38-CLDN bearing hCD3 knock-in mice treated with PBS, 200pmol DVD-WT once every 3 days for total two doses, starting on day 13 after tumor inoculation. n = 5 mice per group.

I, Structural modeling of IL2 (left) and IL2(3E) (right) binding to IL-2Rβ.

J-L, Tumor growth (J), IFN-γ release (K) and body weight (L) in the MC38-CLDN bearing hCD3 knock-in mice treated with PBS, 200pmol DVD, DVD-WT or DVD-3E once every 3 days for total two doses, starting on day 14 after tumor inoculation. n = 5 mice per group.

Data are shown as mean ± SEM and are representative of at least two independent experiments. Statistical analysis was performed using two-tailed unpaired Student's t test (**G**) or one-way ANOVA (K) or two-way ANOVA (**F, J**). ***P < 0.001, ****P < 0.0001, ns, not significant.

Supplemental Figure 4

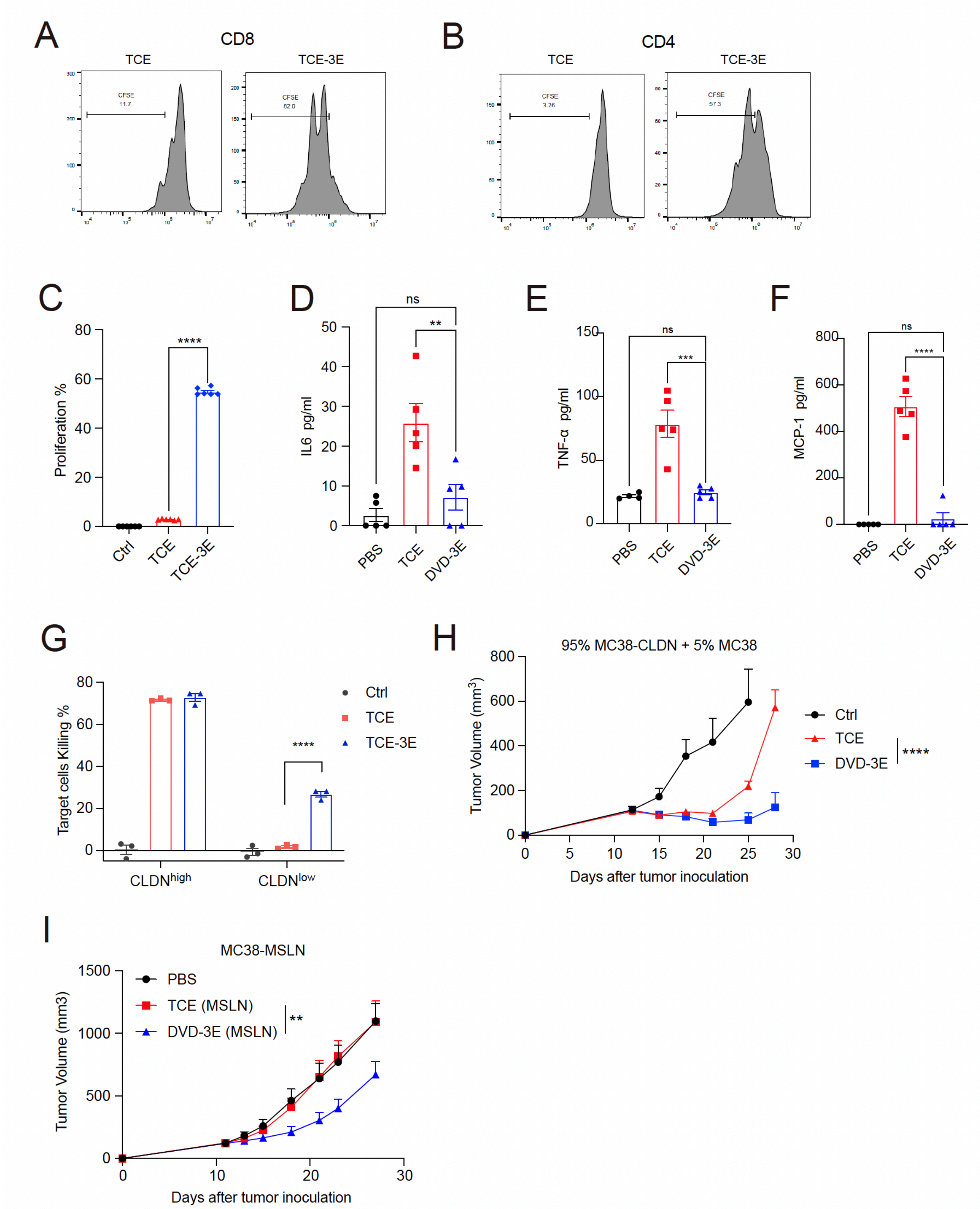

Supplementary Figure 4

A-B, Representative flow cytometry plots showing CFSE-based proliferation of CD8+ (**A**) and CD4+ T (**B**) cells in huPBMC and SNU1-CLDN co-cultures.

C, Quantification of proliferating CD4+ T cells.

D-F, Peripheral blood was collected 48 h after treatment from MC38-CLDN tumor-bearing hCD3 knock-in mice treated with PBS or 200 pmol of TCE or DVD-3E. Serum concentrations of the indicated cytokines were measured by CBA.

G, Cytotoxicity assays using huPBMCs co-cultured with SNU1 cells expressing high or low CLDN levels and treated with TCE or TCE–3E.

H, Tumor growth of mixed MC38-CLDN and MC38 cells (1×10^6 total) inoculated subcutaneously, followed by treatment with TCE or DVD-3E. n = 5 mice per group.

I, MC38-MSLN tumor-bearing hCD3 knock-in mice were randomly assigned to treatment groups and administered intraperitoneally with 200 pmol of TCE (MSLN) or DVD-3E (MSLN) on days 11,13 and 15 after tumor inoculation. n = 5 mice per group.

Data are shown as mean ± SEM and are representative of at least two independent experiments. Statistical analysis was performed using one-way ANOVA (**C-G**) or two-way ANOVA (**H, I**). **P < 0.01, ***P < 0.001, ****P < 0.0001, ns, not significant.

Supplemental Figure 5

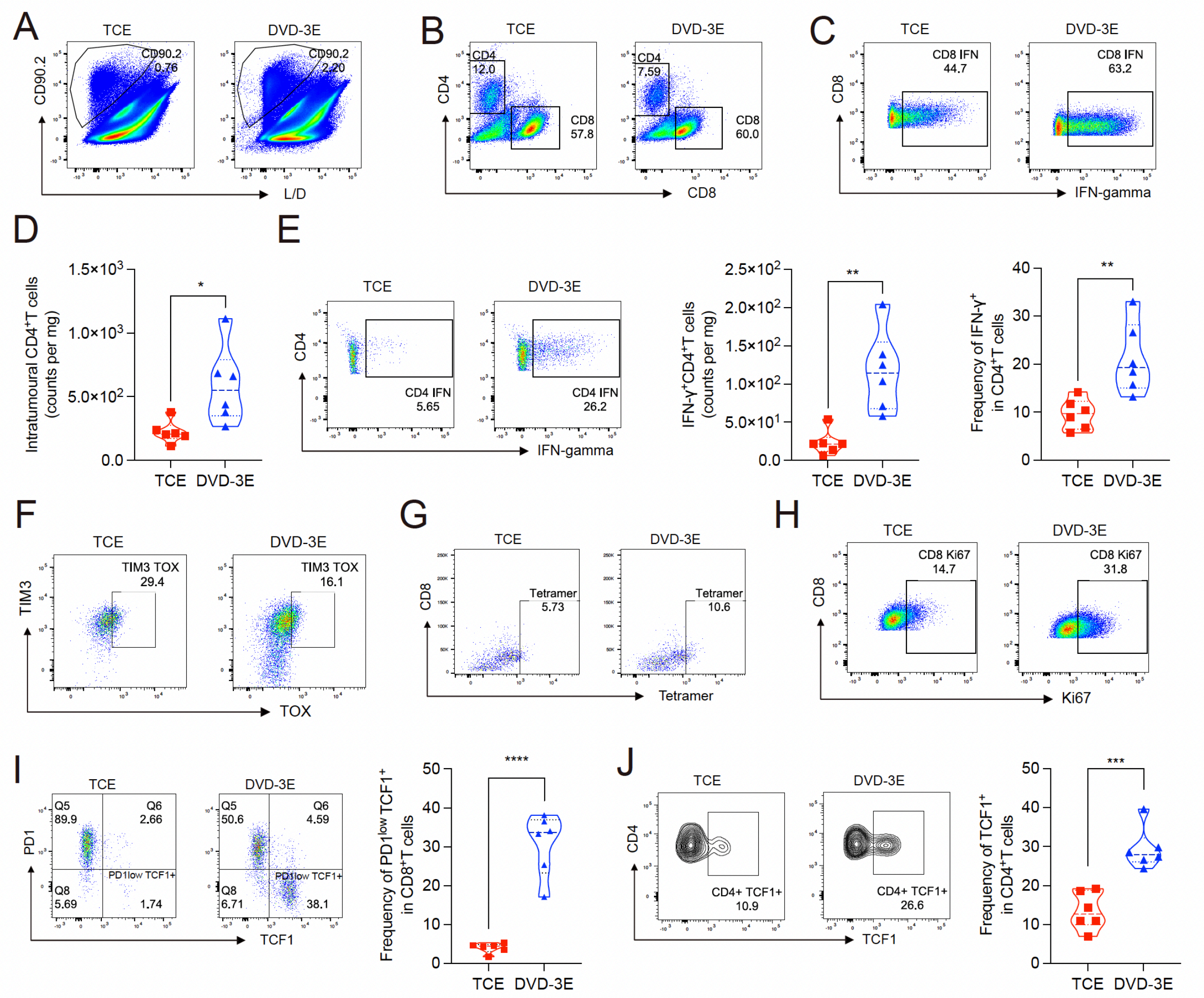

Supplementary Figure 5

hCD3 knock-in mice bearing MC38-CLDN tumors were treated intraperitoneally with 200 pmol TCE or DVD–3E on day 14 and 17. TILs were analyzed 24 h after the second dose. Representative flow cytometry plots show total intratumoral T cells (**A**), $CD8^+$ T and $CD4^+$ T cells (**B**), IFN-γ^+ $CD8^+$ T cells (**C**), quantitative analysis of $CD4^+$ T-cell numbers (**D**), IFN-γ^+ $CD4^+$ T cells (**E**), TOX^+ $CD8^+$ T cells (**F**), $p15E^+$ $Tetramer^+$ $CD8^+$ T cells(**G**), $Ki67^+$ $CD8^+$ T cells (**H**), $TCF1^+$ $PD1^{low}$ T cells (**I**), and $TCF1^+$ $CD4^+$ T cells (**J**). TILs were stimulated *ex vivo* with PMA and ionomycin. n = 6 mice per group.

Data are shown as mean ± SEM and are representative of at least two independent experiments. Each dot represents an individual mouse. Statistical analysis was performed using two-tailed unpaired Student's t test (**D, E , I, J**). $^*P < 0.05$, $^{**}P < 0.01$, $^{***}P < 0.001$, $^{****}P < 0.0001$.

Supplemental Figure 6

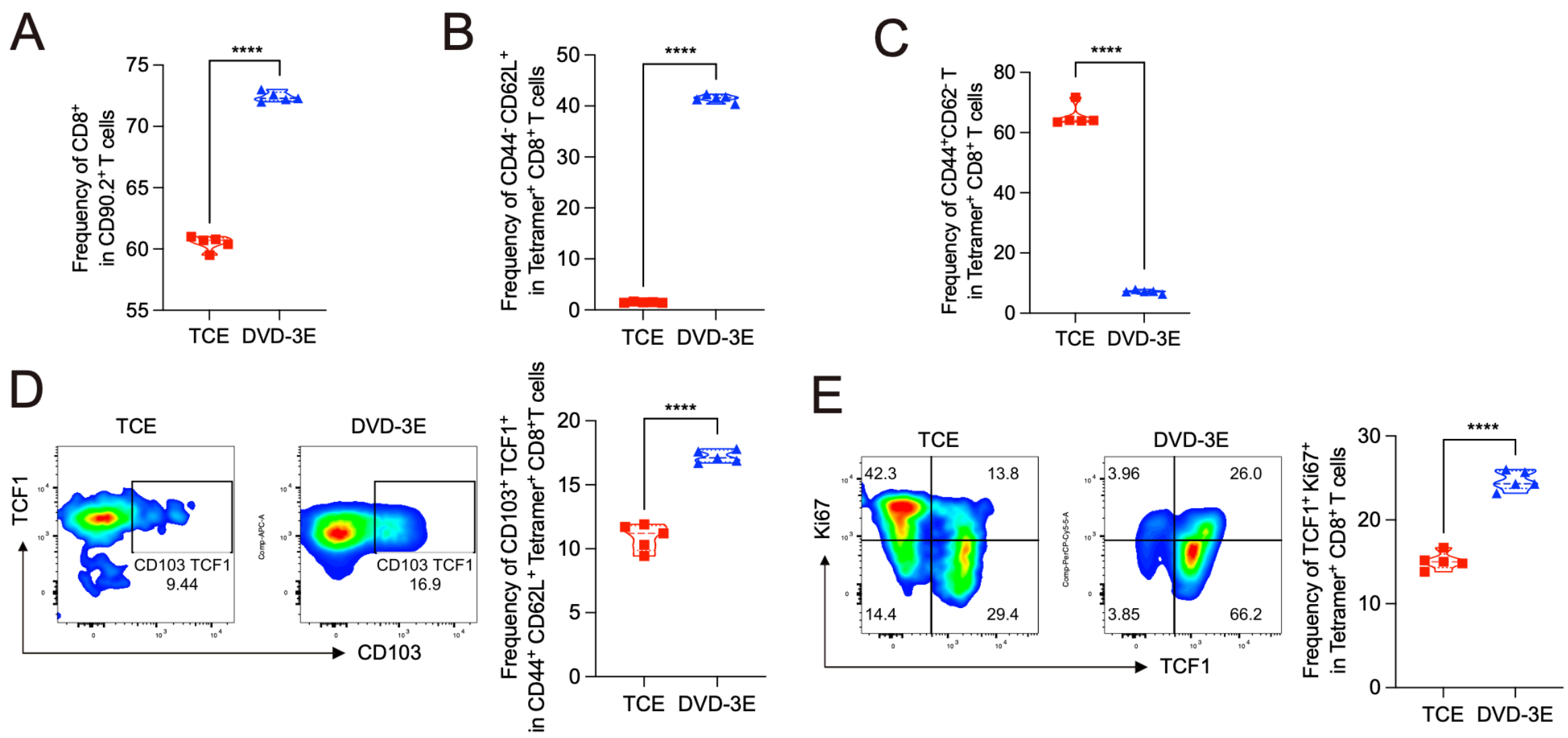

Supplementary Figure 6

A-E. Frequencies of CD8$^+$ T cells (**A**), CD44$^-$CD62L$^+$ tetramer$^+$ naïve-like CD8$^+$ T cells (**B**), CD44$^+$CD62L$^-$ tetramer$^+$ effector CD8$^+$ T cells (**C**), CD103$^+$TCF1$^+$ subsets within CD44$^+$CD62L$^+$ tetramer$^+$ memory CD8$^+$ T cells (**D**), and TCF1$^+$Ki67$^+$ subsets of tetramer$^+$ CD8$^+$ T cells (**E**) in draining lymph nodes.

Data are shown as mean ± SEM and are representative of at least two independent experiments. Each dot represents an individual mouse. Statistical analysis was performed using two-tailed unpaired Student's t test (**A-E**). ****P < 0.0001.
