# Supplementary material for: Logic-gated T cell engager with mutant IL-2 potentiates antitumor responses without cytotoxicity in solid tumor models
Source: J Clin Invest. 2026 Nov 16;136(22):e204657. doi: 10.1172/JCI204657 (PMC13767619; doi:10.1172/JCI204657)
Supplement: Supplementary file 2 [file jci-136-204657-s023.pdf]

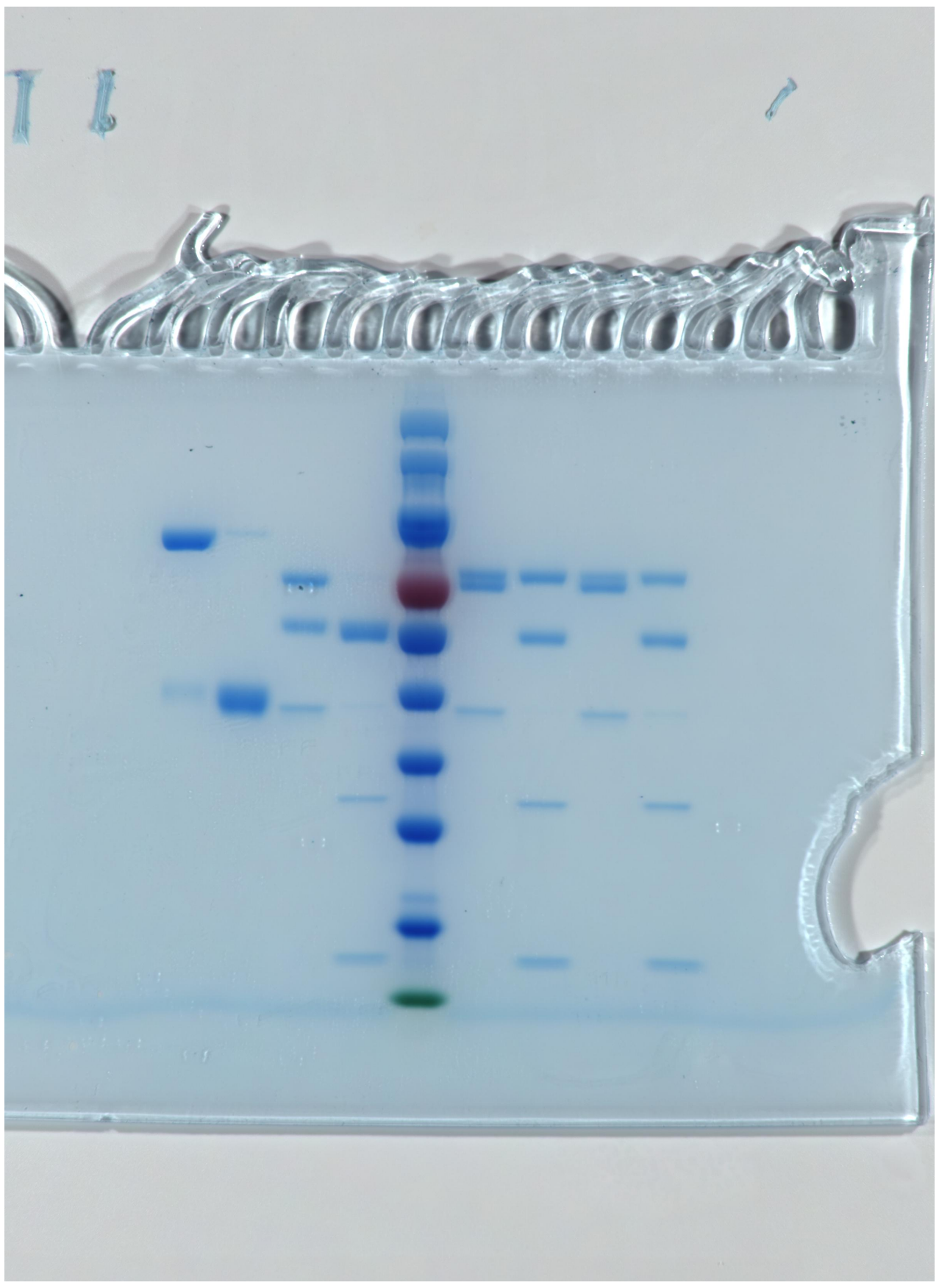

Fig.3G

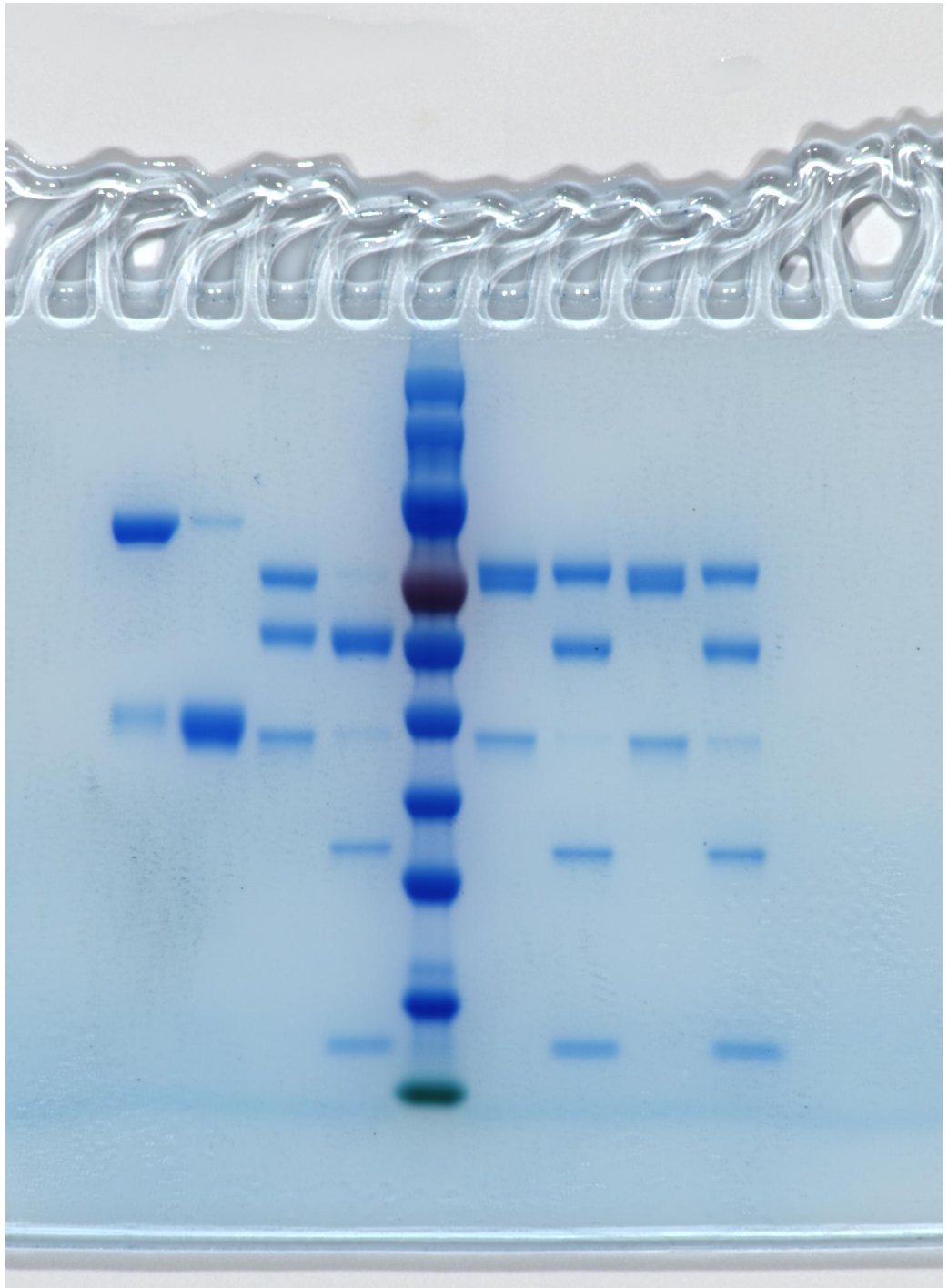

Fig. S2A
